# Interpretable Prediction of Late‐Stage CKM Syndrome Association From Dietary Nutrients in Accelerated Aging Using SHAP and LIME

**DOI:** 10.1002/fsn3.71547

**Published:** 2026-02-17

**Authors:** Hongxiang Tu, Meijie Dai, Yanying Zhu, Min Liang, Mo Shen, Yuehui Chen

**Affiliations:** ^1^ Department of Clinical Laboratory, Key Laboratory of Clinical Laboratory Diagnosis and Translational Research of Zhejiang Province The First Affiliated Hospital of Wenzhou Medical University Wenzhou Zhejiang China

**Keywords:** aging, CKM syndrome, diet, machine learning, phenotypic age, SHAP

## Abstract

The association between habitual dietary nutrient intake and the risk of late‐stage progression of cardiovascular–kidney–metabolic (CKM) syndrome among individuals with accelerated aging remains insufficiently understood. Data were obtained from seven cycles (2005–2018) of the U.S. National Health and Nutrition Examination Survey (NHANES). Six machine learning models were developed to predict late‐stage CKM progression. SHapley Additive exPlanations (SHAP) and Local Interpretable Model‐Agnostic Explanations (LIME) were applied to quantify the relative contributions of individual dietary nutrients to disease risk. Among the evaluated machine learning models, LightGBM and Random Forest demonstrated the highest predictive performance. Time‐series validation further indicated stable model performance across survey cycles. SHAP analysis showed that, when demographic characteristics and dietary intake were jointly incorporated, the strongest negative contributors to late‐stage CKM risk were vitamin B12 (0.011), selenium (0.009), sodium (0.008), moisture (0.008), vitamin B6 (0.008), and vitamin E (0.007). When analyses were restricted to dietary nutrients alone, the leading negative contributors were moisture (0.0597), sodium (0.0368), caffeine (0.0251), niacin (0.0192), vitamin D (0.0191), selenium (0.0188), vitamin B12 (0.0177), and lutein + zeaxanthin (0.0166). Dietary nutrient mixtures are inversely associated with the risk of late‐stage CKM progression in individuals with accelerated aging. LightGBM and Random Forest models achieved superior predictive accuracy. Selenium, sodium, and moisture emerged as prominent protective contributors.

## Graphical abstract

Dietary nutrient mixtures are inversely associated with the risk of late‐stage CKM progression in individuals with accelerated aging. LightGBM and Random Forest demonstrated the highest predictive accuracy. Selenium, sodium, and moisture were among the most notable protective contributors.

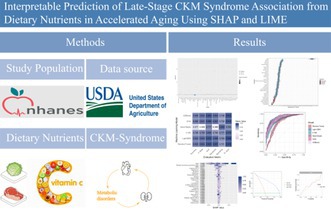

**Abbreviations**
AHA: American Heart Association
ANOVA: analysis of variance
AUC‐PR: area under the precision‐recall curve
AUC‐ROC: area under the receiver operating characteristic curve
BMI: Body Mass Index
CKD: chronic kidney disease
CKM: cardiovascular–kidney–metabolic
CRP: C‐reactive protein
CVD: cardiovascular disease
DCA: Decision Curve Analysis
HDL: high‐density lipoprotein
KNN: k‐nearest neighbors
LDL: low‐density lipoprotein
LightGBM: light gradient boosting machine
LIME: local interpretable model‐agnostic explanations
MEC: mobile examination center
MICE: multiple imputation by chained equations
NCHS: National Center for Health Statistics
NHANES: National Health and Nutrition Examination Survey
PIR: family income‐to‐poverty ratio
SD: standard deviation
SHAP: SHapley Additive exPlanations
SMOTE: synthetic minority oversampling technique
SVM: support vector machine
VIF: variance inflation factor
XGBoost: extreme gradient boosting

## 1 Introduction

The American Heart Association recently defined cardiovascular–kidney–metabolic (CKM) syndrome as an interconnected pathophysiological continuum linking cardiovascular disease (CVD), chronic kidney disease (CKD), and metabolic disorders, including type 2 diabetes and obesity (Ndumele et al. 2023). This multisystem condition represents a major public health challenge because of its high prevalence and strong associations with morbidity and mortality, particularly among older adults (Aggarwal et al. 2024). Approximately one‐third of adults in the United States exhibit three or more CKM risk factors, contributing substantially to rising healthcare expenditures and reduced life expectancy (Ferdinand 2024). As the global population continues to age, identifying determinants that drive progression from early to late stages of CKM syndrome has become increasingly important for effective prevention and risk stratification.

Aging is characterized by progressive physiological decline and increased susceptibility to chronic diseases. Accelerated aging, defined as a biological age that exceeds chronological age, is associated with elevated risks of multiple conditions encompassed within CKM syndrome (Levine et al. 2018). Phenotypic age acceleration, calculated using clinical biomarkers such as creatinine, glucose, and C‐reactive protein (CRP), has emerged as a robust predictor of adverse health outcomes, including cardiovascular events and all‐cause mortality (Zeng et al. 2025; Chaturvedi et al. 2025). Individuals with positive phenotypic age acceleration demonstrate significantly higher risks of CVD, CKD, and type 2 diabetes, highlighting its relevance to CKM progression (Tian et al. 2025; Yao et al. 2025). Consequently, phenotypic age acceleration provides a valuable framework for identifying individuals at heightened risk of rapid disease advancement.

Dietary nutrients play a critical role in modulating inflammation and oxidative stress, two fundamental mechanisms underlying both aging and CKM syndrome (Griffiths et al. 2016). Nutrients such as vitamin C, vitamin E, β‐carotene, and omega‐3 fatty acids exhibit antioxidant and anti‐inflammatory properties that may reduce the risk of chronic diseases (Maleki et al. 2019). For example, omega‐3 fatty acids derived from fatty fish have been shown to lower inflammatory markers and improve lipid profiles in older adults, thereby supporting cardiovascular health (Manson, Cook, Lee, et al. 2019a). Similarly, antioxidants abundant in fruits and vegetables, including vitamin C and polyphenols, may mitigate oxidative stress and slow renal and metabolic deterioration (Fekete et al. 2023). Dietary fiber from whole grains and legumes has also been associated with reduced systemic inflammation and improved metabolic outcomes (Mumme et al. 2022). Together, these findings underscore the potential of dietary modulation to influence multiple components of CKM syndrome.

Despite substantial evidence supporting the roles of individual nutrients in cardiovascular, renal, and metabolic health, the combined effects of dietary nutrient mixtures on CKM progression—particularly among individuals with accelerated aging—remain insufficiently characterized. Inflammation and oxidative stress are central to CKM pathophysiology and represent plausible pathways through which nutrients may exert synergistic protective effects (Sebastian et al. 2024). For instance, folate and magnesium have been reported to attenuate systemic inflammation and potentially slow disease progression in metabolically vulnerable populations (Piccoli et al. 2023; Nannini et al. 2019). However, the interactive and cumulative effects of multiple nutrients within this framework have not been comprehensively examined, leaving an important gap in the current literature.

To address this gap, we leveraged NHANES data—a nationally representative U.S. dataset containing detailed dietary intake, biomarker, and health information—to investigate the associations between dietary nutrients (including carbohydrates, dietary fiber, vitamins, minerals, and fatty acids) and the risk of late‐stage CKM progression in individuals with accelerated aging.

## 2 Materials and Methods

### 2.1 Study Population

**FIGURE 1:**
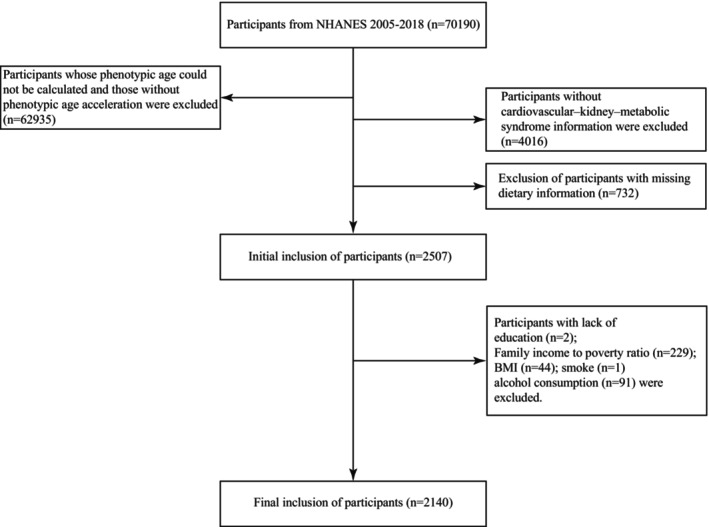
Flowchart of participant inclusion.

The National Health and Nutrition Examination Survey (NHANES), conducted by the National Center for Health Statistics (NCHS), evaluates the health and nutritional status of noninstitutionalized U.S. civilians. Data from the 2005–2018 survey cycles were used in this study, comprising a total of 70,190 participants. Strict exclusion criteria were applied, resulting in the removal of 68,050 individuals who did not meet the study requirements. These exclusions included: (1) inability to calculate phenotypic age or absence of phenotypic age acceleration (*n* = 62,935); (2) missing information on CKM syndrome status or participants classified as CKM stage 0 (*n* = 4,016); (3) lack of dietary nutrient data (*n* = 732); and (4) missing data on education, PIR, BMI, smoking, or drinking status (*n* = 367). Ultimately, 2140 participants were included in the final analysis (Figure 1).

### 2.2 Assessment of Phenotypic Age and PhenoAgeAccel

Phenotypic age was calculated using the formula proposed by Levine et al. (Levine 2013), integrating nine biomarkers—albumin, creatinine, glucose, log‐transformed CRP, lymphocyte percentage, mean cell volume, red cell distribution width, alkaline phosphatase, and white blood cell count—together with chronological age. These biomarkers were selected via a Cox proportional hazards elastic net model with 10‐fold cross‐validation. PhenoAgeAccel was defined as the residual from regressing phenotypic age on chronological age. Negative values indicated biologically younger status, whereas positive values reflected accelerated aging. The full calculation formula is provided below (Levine 2013):

$$\text{Phenotypic}\;\mathrm{Age}=141.50+\frac{\ln\left[-0.00553\times \ln\left(1-M\right)\right]}{0.09165}$$

where

$$M=1-\exp\left(\frac{-1.51714\times \exp\left(\mathrm{xb}\right)}{0.0076927}\right)$$

$$\begin{array}{c} \mathrm{xb}=-19.907+0.0804\times C\text{hronologicalAge}-0.0336\\ \times \text{Albumin}+0.0095\times \text{Creatinine}+0.1953\times \text{Glucose}\\ +0.0954\times \ln\left(\mathrm{CRP}\right)-0.0120\times \text{LymphocytePercent}\\ +0.0268\times \text{MeanCellVolume}+0.3306\times \text{RedCellDistributionWidth}\\ +0.00188\times \text{AlkalinePhosphatase}+0.0554\times \text{WhiteBloodCellCount} \end{array}$$

### 2.3 Assessment of Dietary Nutrients

Dietary intake was assessed using two nonconsecutive 24‐h recalls. The first interview was conducted in person at the Mobile Examination Center (MEC), and the second was conducted by telephone several days later. Data from both recalls were combined to estimate usual nutrient intake. Nutrient intakes were energy‐adjusted using the residual method.

### 2.4 Diagnosis of CKM Syndrome

CKM syndrome was staged according to the American Heart Association (AHA) framework (Ndumele et al. 2023): Stage 0: No risk factors; all indicators normal; Stage 1: Metabolic precursor stage—isolated obesity or prediabetes; Stage 2: Metabolic dysregulation—≥ 1 additional metabolic abnormality or CKD; Stage 3: Subclinical cardiovascular stage—metabolic abnormalities or CKD plus subclinical CVD; Stage 4: Clinical cardiovascular stage—metabolic abnormalities or CKD with clinically manifest CVD. For analysis, stages 1–2 were grouped as early CKM and stages 3–4 as late CKM. The specific NHANES variables and thresholds used for staging are detailed in Table S1.

### 2.5 Covariates

Covariates included sociodemographic and lifestyle characteristics: age, sex, race/ethnicity (Mexican American, Other Hispanic, Non‐Hispanic White, Non‐Hispanic Black, Other), education level (< 9th grade, 9–11th grade, high school diploma/GED, some college/AA degree, ≥ college graduate), PIR, BMI, smoking status, and drinking status. Smoking status: Non‐smokers: never smoked or quit > 1 year ago; Current smokers: smoked in the past 30 days for more than 1 day, smoked upon waking, or smoked > 2 cigarettes daily after quitting. Drinking status: Non‐drinkers: < 12 lifetime drinks; Current drinkers: ≥ 12 drinks/year or > 6 drinks in the past 12 months. BMI: calculated as weight (kg) divided by height squared (m^2^).

### 2.6 Feature Preprocessing and Selection for Machine Learning

A total of 54 features (49 continuous, 5 categorical) were included. Variables with correlation coefficients > 0.9 were removed to reduce collinearity. Class imbalance was addressed using the Synthetic Minority Oversampling Technique (SMOTE), which generates synthetic minority samples by selecting k‐nearest neighbors and interpolating along random directions (Figure S1). All features were standardized before model training. Feature selection was performed using the Boruta algorithm, a random forest–based method that creates “shadow features” to evaluate feature importance. Feature rankings were compared between original and shadow features across 500 iterations until stability was achieved. Only features classified as “confirmed” were retained for model development.

### 2.7 Statistical Analyses

All analyses followed NHANES analytic guidelines. Continuous variables are expressed as means ± standard deviation (SD), and categorical variables as counts and percentages. Group differences were assessed using chi‐square tests for categorical variables and Student's *t*‐tests for continuous variables.

For machine learning, the dataset was split into training (70%) and validation (30%) sets to avoid overfitting. Six algorithms were implemented via the MLR3 framework: Random Forest, LightGBM, k‐Nearest Neighbors (KNN), Naive Bayes, Support Vector Machine (SVM), and XGBoost. Model performance was evaluated using six metrics: accuracy, F‐beta score, AUC‐ROC, sensitivity, specificity, and AUC‐PR, with AUC‐ROC as the primary metric. Ten‐fold cross‐validation was used to improve robustness, and performance differences among models were assessed using ANOVA and Kruskal–Wallis tests. Nested cross‐validation was used to prevent data leakage. No additional multiple comparison corrections (e.g., Bonferroni or FDR) were applied, as the Boruta algorithm and cross‐validation mitigated false positives.

### 2.8 Model Interpretation

The best‐performing model was interpreted using SHAP (SHapley Additive exPlanations) as the primary method; LIME results are in Supporting Information. SHAP quantifies each feature's contribution to predictions by averaging Shapley values across all possible feature combinations. LIME approximates local model behavior with interpretable models (e.g., linear regression) to generate case‐level explanations and enhance transparency.

All statistical analyses were performed using IBM SPSS Statistics (version 24.0) and R (version 4.3.0). A two‐sided *p* < 0.05 was considered statistically significant.

## 3 Results

### 3.1 Classification of Participant Characteristics by CKM Syndrome Stage in Individuals With Accelerated Aging

Table 1 summarizes the baseline characteristics of participants with early‐ and late‐stage CKM syndrome in the accelerated aging cohort. A total of 2140 participants from the 2005–2018 NHANES cycles were included. The mean age was 53.20 years (SD = 17.62), with 773 females (36.12%) and 1367 males (63.88%). Among these, 702 individuals had late‐stage CKM syndrome, with a mean age of 68.86 years (SD = 11.28). Compared with the early‐stage group, participants in the late‐stage group had significantly lower intakes of several dietary nutrients. These included energy (1807.78 vs. 2290.47 kcal, *p* < 0.001), protein (68.22 vs. 85.44 g, *p* < 0.001), carbohydrate (217.79 vs. 271.49 g, *p* < 0.001), total sugar (96.92 vs. 124.16 g, *p* < 0.001), dietary fiber (14.37 vs. 15.50 g, *p* < 0.001), total fat (72.02 vs. 87.81 g, *p* < 0.001), and saturated fatty acids (23.55 vs. 28.76 g, *p* < 0.001).

**TABLE 1:** Baseline characteristics of participants.

Characteristic	Overall *N* = 2140	No CKM syndrome *N* = 1438	CKM syndrome *N* = 702	*p*
Age (year)^a^, Mean ± SD	53.20 ± 17.62	45.56 ± 14.90	68.86 ± 11.28	< 0.001
Sex^b^, *n* (%)				
Female	773 (36.12%)	540 (37.55%)	233 (33.19%)	0.049
Male	1367 (63.88%)	898 (62.45%)	469 (66.81%)	
Race/ethnicity^b^, *n* (%)				
Mexican	285 (13.32%)	221 (15.37%)	64 (9.12%)	< 0.001
Other hispanic	183 (8.55%)	128 (8.90%)	55 (7.83%)	
Non‐Hispanic white	967 (45.19%)	589 (40.96%)	378 (53.85%)	
Non‐Hispanic black	551 (25.75%)	383 (26.63%)	168 (23.93%)	
Other race	154 (7.20%)	117 (8.14%)	37 (5.27%)	
Education^b^, *n* (%)				
< 9th	206 (9.63%)	98 (6.82%)	108 (15.38%)	< 0.001
9–11th	363 (16.96%)	229 (15.92%)	134 (19.09%)	
High school	603 (28.18%)	419 (29.14%)	184 (26.21%)	
Some college	628 (29.35%)	451 (31.36%)	177 (25.21%)	
College graduate	340 (15.89%)	241 (16.76%)	99 (14.10%)	
Family income to poverty ratio^a^, Mean ± SD	2.29 ± 1.53	2.34 ± 1.59	2.18 ± 1.42	0.157
BMI^a^, Mean ± SD	32.38 ± 7.98	32.91 ± 8.06	31.28 ± 7.70	< 0.001
Smoking status^b^, *n* (%)				
No	1488 (69.53%)	946 (65.79%)	542 (77.21%)	< 0.001
Yes	652 (30.47%)	492 (34.21%)	160 (22.79%)	
Drinking status^b^, *n* (%)				
No	223 (10.42%)	136 (9.46%)	87 (12.39%)	0.037
Yes	1917 (89.58%)	1302 (90.54%)	615 (87.61%)	
Energy^a^, Mean ± SD	2132.13 ± 1084.30	2290.47 ± 1149.09	1807.78 ± 850.87	< 0.001
Protein^a^, Mean ± SD	79.79 ± 44.54	85.44 ± 47.58	68.22 ± 34.80	< 0.001
Carbohydrate^a^, Mean ± SD	253.87 ± 138.05	271.49 ± 145.73	217.79 ± 112.59	< 0.001
Total Sugar^a^, Mean ± SD	115.22 ± 88.08	124.16 ± 93.89	96.92 ± 71.44	< 0.001
Dietary fiber^a^, Mean ± SD	15.13 ± 9.81	15.50 ± 9.99	14.37 ± 9.41	0.011
Total Fat^a^, Mean ± SD	82.63 ± 50.28	87.81 ± 53.30	72.02 ± 41.51	< 0.001
Saturated fatty acids^a^, Mean ± SD	27.05 ± 17.70	28.76 ± 18.75	23.55 ± 14.73	< 0.001
Monounsaturated fatty acids^a^, Mean ± SD	29.53 ± 18.87	31.49 ± 20.14	25.54 ± 15.19	< 0.001
Polyunsaturated fatty acids^a^, Mean ± SD	18.36 ± 13.02	19.30 ± 13.68	16.43 ± 11.32	< 0.001
Cholesterol^a^, Mean ± SD	305.13 ± 245.88	318.11 ± 258.16	278.53 ± 216.36	< 0.001
Vitamin E as alpha‐tocopherol^a^, Mean ± SD	7.59 ± 5.98	7.93 ± 6.32	6.90 ± 5.14	< 0.001
Alpha‐tocopherol^a^, Mean ± SD	0.52 ± 3.02	0.53 ± 3.24	0.49 ± 2.49	0.776
Retinol^a^, Mean ± SD	385.82 ± 491.15	391.18 ± 542.65	374.83 ± 363.67	0.840
Vitamin A^a^, Mean ± SD	550.40 ± 612.04	551.62 ± 654.39	547.90 ± 514.96	0.725
Alpha‐carotene^a^, Mean ± SD	318.62 ± 990.53	317.97 ± 1036.49	319.93 ± 889.71	0.611
Beta‐carotene^a^, Mean ± SD	1783.11 ± 3598.89	1734.48 ± 3561.68	1882.72 ± 3674.47	0.496
Beta‐cryptoxanthin^a^, Mean ± SD	69.94 ± 151.57	66.79 ± 139.14	76.39 ± 174.21	0.177
Lycopene^a^, Mean ± SD	5130.75 ± 9681.33	5520.99 ± 10,187.78	4331.37 ± 8501.87	< 0.001
Lutein+zeaxanthin^a^, Mean ± SD	1201.69 ± 2487.02	1245.36 ± 2697.65	1112.25 ± 1985.85	0.952
Thiamin (Vitamin B1) ^a^, Mean ± SD	1.54 ± 0.86	1.61 ± 0.91	1.41 ± 0.74	< 0.001
Riboflavin (Vitamin B2)^a^, Mean ± SD	2.02 ± 1.45	2.10 ± 1.58	1.86 ± 1.12	0.001
Niacin^a^, Mean ± SD	24.95 ± 16.40	27.02 ± 18.11	20.69 ± 11.01	< 0.001
Vitamin B6^a^, Mean ± SD	1.97 ± 1.62	2.12 ± 1.78	1.68 ± 1.15	< 0.001
Total folate^a^, Mean ± SD	368.61 ± 234.70	386.94 ± 248.13	331.06 ± 199.41	< 0.001
Folic acid^a^, Mean ± SD	169.38 ± 169.27	179.61 ± 181.34	148.44 ± 139.14	< 0.001
Food folate^a^, Mean ± SD	199.41 ± 132.11	207.51 ± 138.67	182.81 ± 115.86	< 0.001
Folate (DFE) ^a^, Mean ± SD	487.06 ± 339.86	512.55 ± 361.01	434.85 ± 285.00	< 0.001
Total choline^a^, Mean ± SD	333.72 ± 210.74	349.69 ± 225.54	301.01 ± 172.19	< 0.001
Vitamin B12^a^, Mean ± SD	5.05 ± 7.93	5.50 ± 9.26	4.12 ± 3.89	< 0.001
Added vitamin B12^a^, Mean ± SD	0.83 ± 2.79	0.90 ± 2.97	0.69 ± 2.38	0.350
Vitamin C^a^, Mean ± SD	73.84 ± 99.32	76.52 ± 107.27	68.34 ± 80.40	0.580
Vitamin K^a^, Mean ± SD	92.14 ± 125.26	94.30 ± 132.09	87.74 ± 109.89	0.149
Calcium^a^, Mean ± SD	898.40 ± 620.59	947.70 ± 641.51	797.43 ± 562.46	< 0.001
Phosphorus^a^, Mean ± SD	1313.39 ± 706.12	1394.38 ± 740.37	1147.49 ± 597.29	< 0.001
Magnesium^a^, Mean ± SD	275.48 ± 146.87	289.36 ± 153.22	247.05 ± 128.42	< 0.001
Iron^a^, Mean ± SD	14.17 ± 8.96	14.67 ± 9.17	13.16 ± 8.44	< 0.001
Zinc^a^, Mean ± SD	11.27 ± 12.72	12.10 ± 14.92	9.56 ± 5.76	< 0.001
Copper^a^, Mean ± SD	1.19 ± 1.23	1.26 ± 1.45	1.03 ± 0.56	< 0.001
Sodium^a^, Mean ± SD	3509.12 ± 1943.22	3748.07 ± 2008.42	3019.65 ± 1701.83	< 0.001
Potassium^a^, Mean ± SD	2505.50 ± 1319.03	2586.00 ± 1376.07	2340.60 ± 1177.57	< 0.001
Selenium^a^, Mean ± SD	111.77 ± 69.45	119.22 ± 75.17	96.53 ± 52.81	< 0.001
Caffeine^a^, Mean ± SD	168.49 ± 250.24	163.77 ± 233.55	178.16 ± 281.28	0.061
Theobromine^a^, Mean ± SD	33.44 ± 72.29	35.31 ± 76.29	29.61 ± 63.17	0.574
Alcohol^a^, Mean ± SD	10.97 ± 30.03	13.69 ± 33.71	5.41 ± 19.38	< 0.001
Moisture^a^, Mean ± SD	2918.15 ± 1615.30	3116.74 ± 1659.34	2511.36 ± 1438.98	< 0.001
Vitamin D^a^, Mean ± SD	4.19 ± 5.86	4.22 ± 6.19	4.11 ± 5.12	0.258

Abbreviation: SD, standard deviation.

^a^ Student's *t*‐test.

^b^ Chi‐square test.

### 3.2 Feature Selection in Machine Learning Models

**FIGURE 2:**
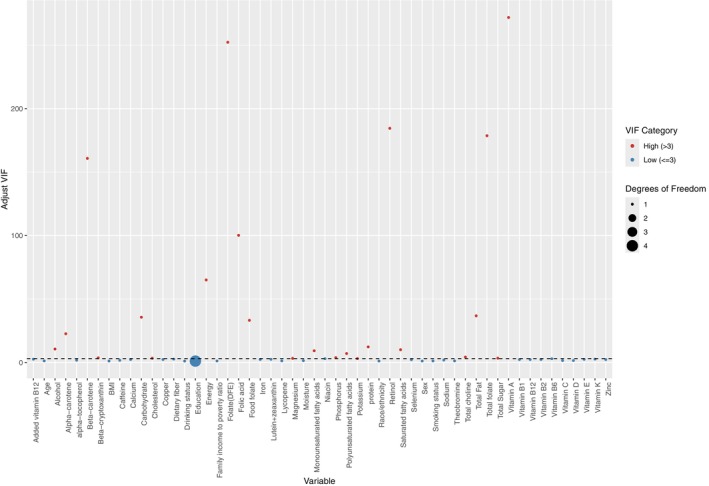
Variance inflation factors of different features. Red: Collinearity present; Blue: No collinearity.

Variance inflation factor (VIF) analysis identified variables with adjusted VIF > 3 as collinear (Figure 2), leading to the exclusion of 23 features, including alcohol, alpha‐carotene, beta‐carotene, beta‐cryptoxanthin, carbohydrate, cholesterol, energy, folate (DFE), folic acid, food folate, magnesium, monounsaturated fatty acids, phosphorus, polyunsaturated fatty acids, potassium, protein, retinol, saturated fatty acids, total choline, total fat, total folate, total sugar, and vitamin A.

**FIGURE 3:**
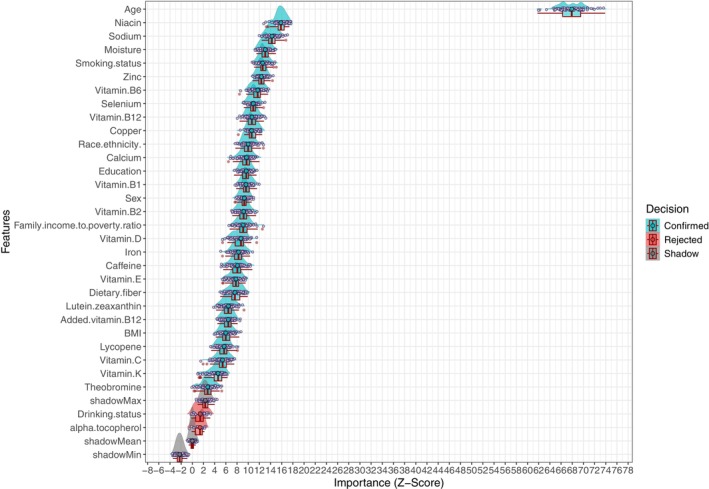
Ridge plot showing variable selection results from the Boruta algorithm.

After removing collinear variables, the Boruta algorithm identified 29 “confirmed” variables associated with CKM progression risk: seven demographic factors and 22 dietary nutrients. These included age, niacin, sodium, moisture, smoking status, zinc, vitamin B6, selenium, vitamin B12, copper, race/ethnicity, calcium, education, vitamin B1, sex, vitamin B2, PIR, vitamin D, iron, caffeine, vitamin E, dietary fiber, lutein+zeaxanthin, added vitamin B12, BMI, lycopene, vitamin C, vitamin K, and theobromine. Drinking status and alpha‐tocopherol were excluded due to negligible contribution (Figure 3). Figure S2 illustrates *Z*‐score stability across iterations.

### 3.3 Construction and Evaluation of Machine Learning Models

Six machine learning algorithms—Random Forest, LightGBM, KNN, Naive Bayes, SVM, and XGBoost were trained and evaluated using two input sets (Figures 4 and 5): demographic + dietary variables (Tables 2 and 3) and dietary variables only (Tables 4 and 5). Model performance was assessed using AUC‐ROC (Figures 6, 7, 8), AUC‐PR (Figures 9, 10, 11), accuracy (Figure 12), F‐beta score (Figure 13), sensitivity (Figure 14), and specificity (Figure 15).

**FIGURE 4:**
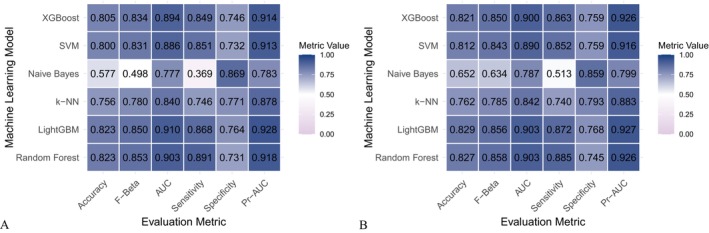
Heatmap comparing the performance of different machine learning models when combining demographic characteristics with dietary nutrients. (A) Training set; (B) Validation set.

**FIGURE 5:**
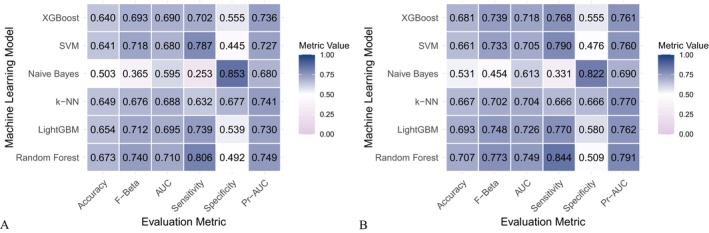
Heatmap comparing the performance of different machine learning models using dietary nutrients alone. (A) Training set; (B) Validation set.

**TABLE 2:** Benchmark comparison results for the training set with combined demographic characteristics and dietary nutrients.

Model	Accuracy	F Beta	Area under the ROC curve	Sensitivity	Specificity	Area under the PR curve
Random Forest	0.823	0.853	0.903	0.891	0.731	0.918
Light GBM	0.823	0.850	0.910	0.868	0.764	0.928
KNN	0.756	0.780	0.840	0.746	0.771	0.878
Naive Bayes	0.577	0.498	0.777	0.369	0.869	0.783
SVM	0.800	0.831	0.886	0.851	0.732	0.913
XGBoost	0.805	0.834	0.894	0.849	0.746	0.914
*p*	< 0.001^a^	< 0.001^a^	< 0.001^b^	< 0.001^a^	< 0.001^a^	< 0.001^a^

^a^ ANOVA test.

^b^ Kruskal–Wallis test.

**TABLE 3:** Benchmark comparison results for the validation set with combined demographic characteristics and dietary nutrients.

Model	Accuracy	F Beta	Area under the ROC curve	Sensitivity	Specificity	Area under the PR curve
Random Forest	0.827	0.858	0.903	0.885	0.745	0.926
Light GBM	0.829	0.856	0.903	0.872	0.768	0.927
KNN	0.762	0.785	0.842	0.740	0.793	0.883
Naive Bayes	0.652	0.634	0.787	0.513	0.859	0.799
SVM	0.812	0.843	0.890	0.852	0.759	0.916
XGBoost	0.821	0.850	0.900	0.863	0.759	0.926
*p*	< 0.001^a^	< 0.001^a^	< 0.001^b^	< 0.001^a^	< 0.001^a^	< 0.001^a^

^a^ ANOVA test.

^b^ Kruskal–Wallis test.

**TABLE 4:** Benchmark comparison results for the training set using dietary nutrients alone.

Model	Accuracy	F Beta	Area under the ROC curve	Sensitivity	Specificity	Area under the PR curve
Random Forest	0.673	0.740	0.710	0.806	0.492	0.749
Light GBM	0.654	0.712	0.695	0.739	0.539	0.730
KNN	0.649	0.676	0.688	0.632	0.677	0.741
Naive Bayes	0.503	0.365	0.595	0.253	0.853	0.680
SVM	0.641	0.718	0.680	0.787	0.445	0.727
XGBoost	0.640	0.693	0.690	0.702	0.555	0.736
*p*	< 0.001^a^	< 0.001^a^	< 0.001^b^	< 0.001^a^	< 0.001^a^	< 0.001^a^

^a^ ANOVA test.

^b^ Kruskal–Wallis test.

**TABLE 5:** Benchmark comparison results for the validation set using dietary nutrients alone.

Model	Accuracy	F Beta	Area under the ROC curve	Sensitivity	Specificity	Area under the PR curve
Random Forest	0.707	0.773	0.749	0.844	0.509	0.791
Light GBM	0.693	0.748	0.726	0.770	0.580	0.762
KNN	0.667	0.702	0.704	0.666	0.666	0.770
Naive Bayes	0.531	0.454	0.613	0.331	0.822	0.690
SVM	0.661	0.733	0.705	0.790	0.476	0.760
XGBoost	0.681	0.739	0.718	0.768	0.555	0.761
*p*	< 0.001^a^	< 0.001^a^	< 0.001^b^	< 0.001^a^	< 0.001^a^	< 0.001^a^

^a^ ANOVA test.

^b^ Kruskal–Wallis test.

**FIGURE 6:**
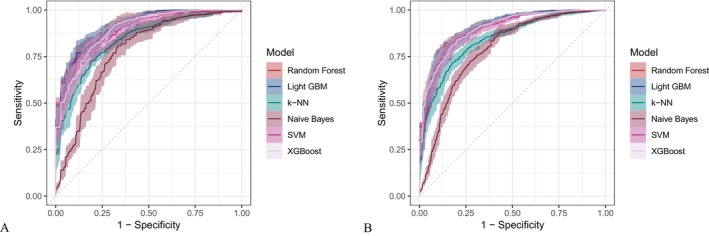
ROC curves of six different machine learning models with combined demographic characteristics and dietary nutrients. (A) Training set; (B) Validation set.

**FIGURE 7:**
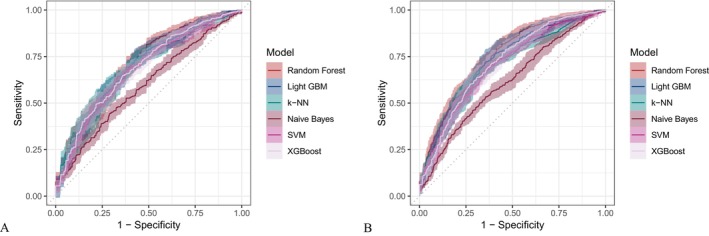
ROC curves of six different machine learning models using dietary nutrients alone. (A) Training set; (B) Validation set.

**FIGURE 8:**
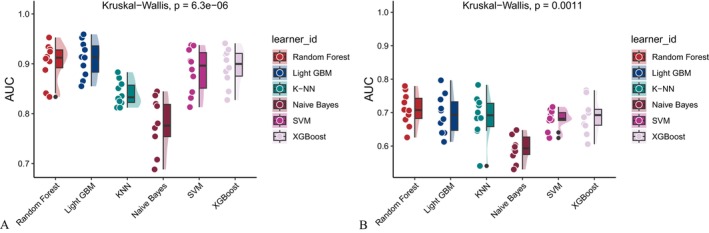
Raincloud plots of area under the curve (AUC) for six different machine learning models. (A) Combined demographic characteristics and dietary nutrients; (B) Dietary nutrients alone.

**FIGURE 9:**
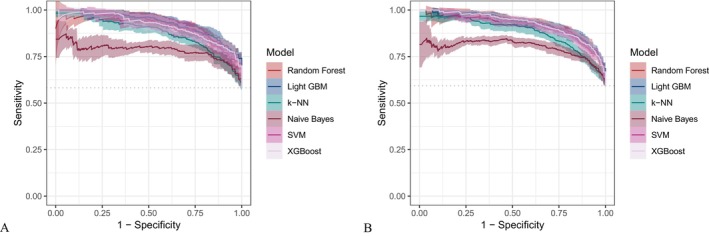
Precision–recall (PR) curves of six different machine learning models with combined demographic characteristics and dietary nutrients. (A) Training set; (B) Validation set.

**FIGURE 10:**
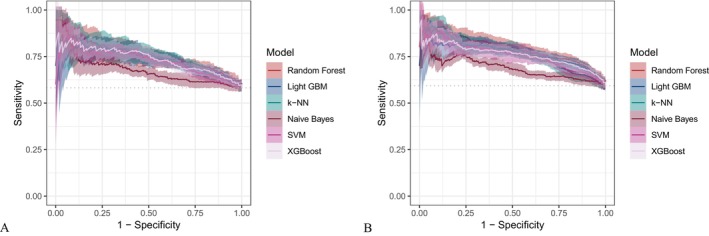
PR curves of six different machine learning models using dietary nutrients alone. (A) Training set; (B) Validation set.

**FIGURE 11:**
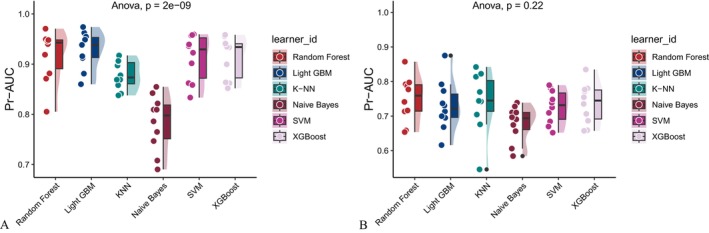
Raincloud plots of area under the precision–recall curve (AUC‐PR) for six different machine learning models. (A) Combined demographic characteristics and dietary nutrients; (B) Dietary nutrients alone.

**FIGURE 12:**
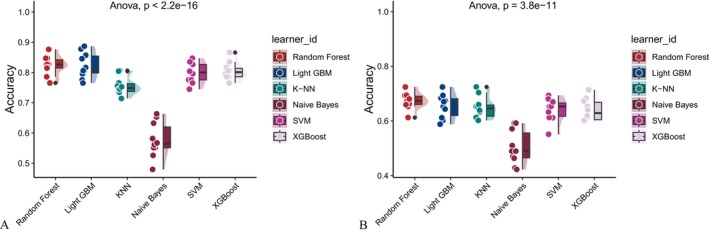
Raincloud plots of accuracy for six different machine learning models. (A) Combined demographic characteristics and dietary nutrients; (B) Dietary nutrients alone.

**FIGURE 13:**
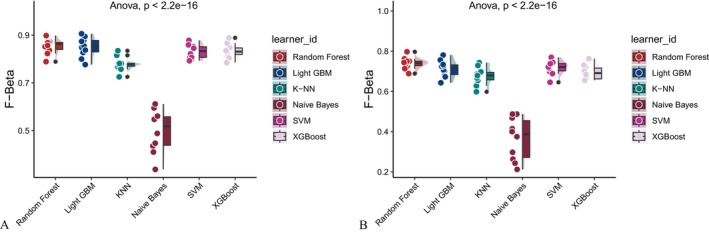
Raincloud plots of F‐beta scores for six different machine learning models. (A) Combined demographic characteristics and dietary nutrients; (B) Dietary nutrients alone.

**FIGURE 14:**
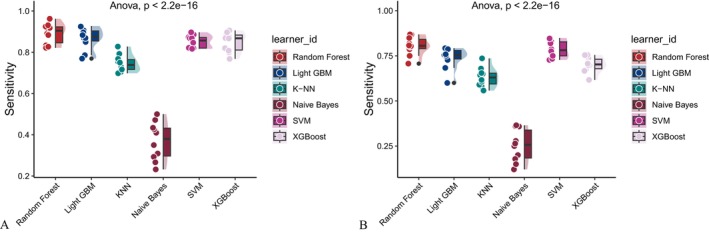
Raincloud plots of sensitivity for six different machine learning models. (A) Combined demographic characteristics and dietary nutrients; (B) Dietary nutrients alone.

**FIGURE 15:**
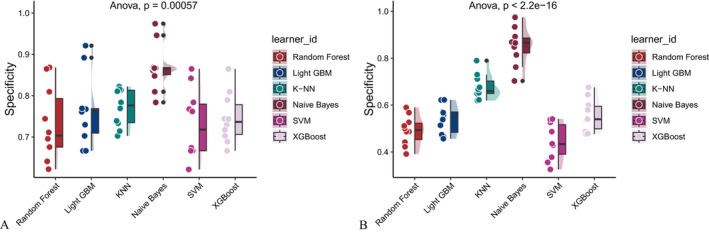
Raincloud plots of specificity for six different machine learning models. (A) Combined demographic characteristics and dietary nutrients; (B) Dietary nutrients alone.

With demographics included, LightGBM demonstrated the best overall performance in both training and validation sets. In the training set, it achieved an accuracy of 0.823, F‐beta of 0.850, AUC‐ROC of 0.910, sensitivity of 0.868, specificity of 0.764, and AUC‐PR of 0.928. Random Forest and XGBoost followed closely (AUC‐ROC: 0.903 and 0.894; AUC‐PR: 0.918 and 0.914, respectively). KNN and SVM performed moderately, whereas Naive Bayes showed the lowest performance (AUC‐ROC: 0.777, AUC‐PR: 0.783) (Table 2). In the validation set, LightGBM again ranked highest (accuracy: 0.829; F‐beta: 0.856; AUC‐ROC: 0.903; sensitivity: 0.872; specificity: 0.768; AUC‐PR: 0.927) (Table 3).

When only dietary variables were included, Random Forest performed best. In the training set, it achieved an accuracy of 0.673, F‐beta of 0.740, AUC‐ROC of 0.710, sensitivity of 0.806, specificity of 0.492, and AUC‐PR of 0.749 (Table 4). In the validation set, its performance improved to an accuracy of 0.707, F‐beta of 0.773, AUC‐ROC of 0.749, sensitivity of 0.844, specificity of 0.509, and AUC‐PR of 0.791 (Table 5). Across models, performance differences were statistically significant (*p* < 0.001).

### 3.4 Explaining Nutrient Contributions Using SHAP and LIME

**FIGURE 16:**
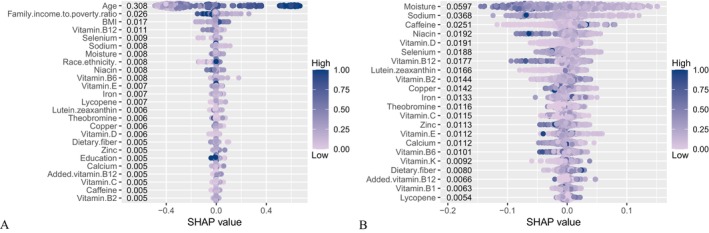
SHAP beeswarm plots showing feature selection results and SHAP values for the best‐performing model. (A) Combined demographic characteristics and dietary nutrients; (B) Dietary nutrients alone.

**FIGURE 17:**
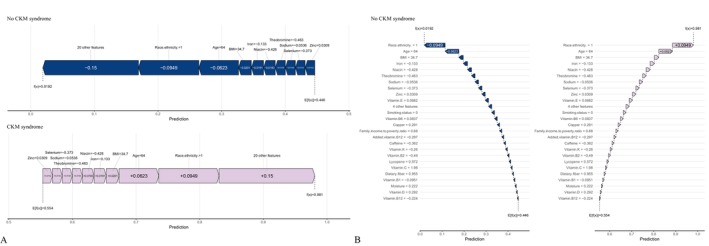
Force and waterfall plots illustrating case‐level prediction results of the best‐performing model with combined demographic characteristics and dietary nutrients. (A) Force plot; (B) Waterfall plot.

**FIGURE 18:**
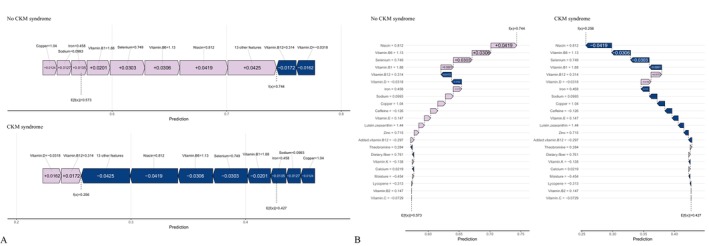
Force and waterfall plots illustrating case‐level prediction results of the best‐performing model using dietary nutrients alone. (A) Force plot; (B) Waterfall plot.

**FIGURE 19:**
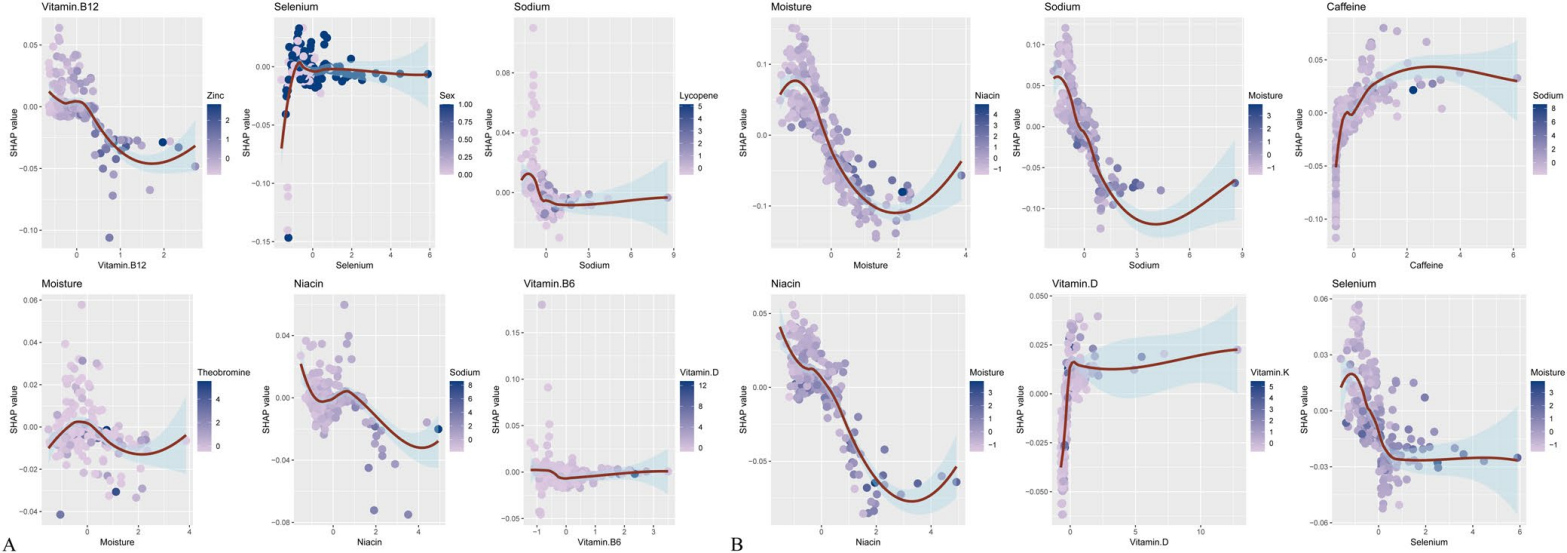
Interaction dependence plots showing the relationships between SHAP values and the top six dietary nutrients features. (A) Combined demographic characteristics and dietary nutrients; (B) Dietary nutrients alone.

SHAP values quantified the contribution of individual features to CKM progression predictions (Figure 16). With demographics included, age (0.308) was the strongest positive contributor. The main negative contributors were vitamin B12 (0.011), selenium (0.009), sodium (0.008), moisture (0.008), vitamin B6 (0.008), and vitamin E (0.007). With dietary variables only, the strongest negative contributors were moisture (0.0597), sodium (0.0368), caffeine (0.0251), niacin (0.0192), vitamin D (0.0191), selenium (0.0188), vitamin B12 (0.0177), and lutein+zeaxanthin (0.0166). Force and waterfall plots (Figures 17, 18) illustrated case‐level feature effects. For instance, with demographics and diet, the baseline probability of no progression was 0.446, which dropped to 0.0192 after accounting for feature effects. With diet alone, the probability increased to 0.744. Interaction dependence plots (Figure 19) revealed relationships between SHAP values and the top six features for each scenario.

LIME analysis provided localized interpretability. With demographics and diet, the predicted probability of no progression was 0.979, influenced most by caffeine (−0.521 to −0.182), vitamin B12 (−0.155 to 0.249), vitamin E (> 0.531), added vitamin B12 (> −0.189), zinc (> 0.288), and moisture (−0.0689 to 0.4617) (Figure S3). With diet alone, the predicted probability was 0.744, with key drivers including niacin (> 0.5649), vitamin B6 (> 0.4736), vitamin B12 (> 0.2615), selenium (> 0.570), iron (−0.0159 to 0.6501), and theobromine (> 0.132) (Figure S4).

### 3.5 Sensitivity Analysis

To evaluate the robustness of the study models, a comprehensive set of sensitivity analyses was performed. First, analyses were repeated in the full population without restricting participants to those with PhenoAgeAccel > 0. In models jointly incorporating demographic characteristics and dietary nutrients, LightGBM consistently remained the best‐performing algorithm. Similarly, in models including only dietary nutrients, random forest continued to demonstrate optimal performance. In both scenarios, changes in AUC‐ROC were less than 0.05 compared with analyses restricted to participants with PhenoAgeAccel > 0 (Figure S5).

Second, VIF–based feature selection was replaced with the residual method, and models were reconstructed using the selected variables. Model performance using the residual method was comparable to that achieved with VIF screening, with AUC‐ROC differences also remaining below 0.05 (Figure S6A–B).

Third, calibration curves and decision curve analysis (DCA) were generated, and the Brier score was calculated to further assess model stability. The calibration curves, together with a Brier score of 0.096, indicated stable performance of the LightGBM model. DCA showed that when the threshold probability ranged from 0.2 to 0.7, the net benefit of the model exceeded 0.2 (Figure S6C–D).

Fourth, participants with pre‐existing CVD or CKD were excluded (*n* = 813), and the models were reconstructed. No substantial differences were observed between the reconstructed and original models. When demographic characteristics and dietary nutrients were jointly considered, LightGBM again exhibited the best performance (Figure S7A–B). To reduce potential bias from complete‐case analysis due to missing data, multiple imputation by chained equations (MICE) was applied, followed by model reconstruction. The performance of machine learning models based on imputed data was comparable to that of the original models (Figure S7C–D).

Finally, time‐series validation was conducted to further confirm model stability. Data from 2005 to 2014 were used as the training set, and data from 2015 to 2018 served as the validation set. Under models jointly incorporating demographic characteristics and dietary nutrients, LightGBM again achieved the best performance, confirming the robustness of the model (Figure S8).

## 4 Discussion

Using data from NHANES and multiple machine learning approaches, this study examined the associations between dietary nutrient intake and the risk of progression to late‐stage CKM syndrome among individuals with accelerated aging. After adjustment for potential confounders, higher overall nutrient intake was consistently associated with a lower risk of CKM progression. When demographic characteristics and dietary nutrients were jointly modeled, LightGBM demonstrated the strongest predictive performance, whereas Random Forest achieved the highest accuracy when dietary variables alone were considered. Model interpretability using SHAP analysis further identified key nutrients associated with reduced CKM risk. In the combined demographics‐and‐diet model, the strongest negative contributors included vitamin B12 (0.011), selenium (0.009), sodium (0.008), moisture (0.008), vitamin B6 (0.008), and vitamin E (0.007). In the diet‐only model, the leading contributors were moisture (0.0597), sodium (0.0368), caffeine (0.0251), niacin (0.0192), vitamin D (0.0191), selenium (0.0188), vitamin B12 (0.0177), and lutein + zeaxanthin (0.0166).

Many of the identified nutrients have well‐established roles in preventing chronic diseases central to CKM syndrome. Vitamins B12 and B6 are essential regulators of homocysteine metabolism, and elevated homocysteine levels are a recognized risk factor for CVD (Kataria et al. 2021). Meta‐analyses of randomized controlled trials indicate that B‐vitamin supplementation effectively reduces homocysteine concentrations and may lower cardiovascular risk (Jenkins et al. 2021). Selenium, an essential trace element with antioxidant properties, has been associated with reduced coronary heart disease incidence in some observational studies (Wang et al. 2023), although evidence from systematic reviews remains inconsistent (Rayman 2020). Vitamin E, another potent antioxidant, mitigates oxidative stress and inflammation—key mechanisms in cardiovascular and metabolic disorders (Giosuè et al. 2022)—yet supplementation trials have yielded mixed findings (Myung et al. 2013).

Niacin (vitamin B3) favorably influences lipid metabolism by increasing high‐density lipoprotein cholesterol and lowering low‐density lipoprotein cholesterol and triglycerides, thereby supporting cardiovascular health (Hrubša et al. 2022). Vitamin D deficiency has been linked to increased risks of CVD, type 2 diabetes, and CKD, and observational evidence suggests potential benefits of adequate vitamin D status or supplementation (Zhao et al. 2024; Manson, Cook, Lee, et al. 2019b; Ou et al. 2025). Adequate hydration, reflected by moisture intake, supports renal function and may reduce disease exacerbation associated with dehydration (Clark et al. 2016). Moderate caffeine consumption has been associated with lower risks of type 2 diabetes and CVD, potentially through anti‐inflammatory and insulin‐sensitizing mechanisms (Ding et al. 2014). Lutein and zeaxanthin, carotenoids traditionally recognized for ocular health, also exhibit anti‐inflammatory and antioxidant effects that may contribute to improved cardiovascular function (Scripsema et al. 2015). Although dietary fiber was not among the top SHAP‐ranked features in this study, prior research consistently demonstrates its role in reducing inflammation and improving metabolic health in older adults (Shivakoti et al. 2022), reinforcing the broader benefits of nutrient‐dense dietary patterns.

An unexpected finding was the inverse association between sodium intake and the risk of CKM progression, which contrasts with extensive evidence linking high sodium intake to hypertension, CVD, and kidney dysfunction (Mente et al. 2016). This observation may reflect a J‐shaped association, wherein both very low and very high sodium intakes are associated with increased morbidity and mortality, as reported in several meta‐analyses (Graudal et al. 2014). Sensitivity analyses excluding participants with cardiovascular or CKD yielded consistent results. In this cohort, sodium intake may have fallen within a range that did not confer excess risk, or the association may reflect altered sodium handling, dietary patterns, or physiological adaptations among individuals with accelerated aging (Cogswell et al. 2018). Interactions with other nutrients, particularly potassium, could also influence sodium's impact, as higher potassium intake can mitigate sodium's hypertensive effects (Binia et al. 2015). These results emphasize the importance of considering nutrient interactions, physiological status, and population‐specific factors when interpreting dietary associations in CKM (Esposito et al. 2024). Future analyses should incorporate interaction terms or network models to explore synergies and antagonisms among nutrients (Tessier et al. 2025). Higher potassium intake can mitigate sodium's hypertensive effects, and such a balance may exist in our study population. Reverse causation is another possibility, as individuals with advanced CKM may have been advised to restrict sodium, resulting in lower reported intake among those with more severe disease (McCarron et al. 2013).

Caffeine exhibited similarly complex associations. While moderate consumption is generally linked to cardiometabolic benefits, excessive intake may elevate blood pressure and pose risks for certain subgroups (Haghighatdoost et al. 2023). These results emphasize the importance of considering nutrient interactions, physiological status, and population‐specific factors when interpreting dietary associations in CKM. From a mechanistic perspective, our results support the hypothesis that anti‐inflammatory and antioxidant nutrients may attenuate disease progression by reducing oxidative stress and systemic inflammation—core pathophysiological processes in CKM (Furman et al. 2019; Zuin et al. 2025). However, this study lacks experimental validation at the cellular, animal, or clinical sample level, limiting our ability to elucidate underlying biological mechanisms. Future studies should integrate multi‐omics or experimental approaches to confirm these associations, including interaction terms or network models (Chen et al. 2025). Identifying these protective nutrients is consistent with nutritional epidemiology models highlighting the role of micronutrients in healthy aging. The sodium finding challenges current guidelines recommending broad sodium restriction for CVD and kidney disease prevention (Whelton et al. 2018) and suggests that sodium's effects may be more context‐dependent, particularly among individuals with accelerated aging (Ge et al. 2025).

The application of machine learning models combined with SHAP analysis highlights the utility of advanced analytical techniques for identifying complex, nonlinear nutrient–disease relationships that may not be captured by traditional statistical methods (Bi et al. 2019). These approaches offer promising tools for advancing precision nutrition strategies in CKM prevention and management.

Several limitations should be acknowledged. First, the cross‐sectional design of NHANES precludes causal inference. Second, reverse causation cannot be excluded, as dietary behaviors may change following CKM diagnosis or progression. Third, dietary intake was assessed using 24‐h recalls, which are subject to recall and reporting bias and may not accurately reflect habitual intake. Fourth, phenotypic age acceleration, although validated, may not fully capture all dimensions of biological aging. Fifth, the findings may not be generalizable beyond the U.S. population. Sixth, residual confounding from unmeasured factors such as medication use or socioeconomic status may persist. Seventh, the machine learning models did not account for NHANES sampling weights, stratification, or clustering, potentially limiting population‐level inference. Eighth, external validation was not available, and although time‐series validation was performed, independent cohort validation is required. Finally, SHAP values represent associations rather than causal effects and should be interpreted cautiously.

Despite these limitations, this study provides novel evidence linking specific dietary nutrients to reduced risk of late‐stage CKM progression among individuals with accelerated aging. The integration of machine learning and explainable AI methods offers a valuable framework for future longitudinal investigations and the development of targeted dietary interventions.

## 5 Conclusion

In conclusion, our findings underscore the pivotal role of nutrition in chronic disease prevention and management and demonstrate the potential of machine learning in nutritional epidemiology. Longitudinal studies are needed to confirm these associations, elucidate underlying mechanisms, and refine dietary recommendations tailored to individuals with CKM and accelerated aging, including public health strategies for nutrient optimization.

## Author Contributions

**Hongxiang Tu:** conceptualization, formal analysis, investigation, visualization, writing – review and editing. **Meijie Dai:** data curation, formal analysis, validation, visualization, writing – original draft. **Yanying Zhu:** data curation, validation, writing – original draft. **Min Liang:** supervision, project administration. **Mo Shen:** formal analysis, methodology, data interpretation, writing – review and editing. **Yuehui Chen:** conceptualization, methodology, writing – review and editing, writing – original draft, funding acquisition.

## Funding

This research was backed up by the Key Laboratory of Clinical Laboratory Diagnosis and Translational Research of Zhejiang Province (2022E10022).

## Ethics Statement

This study was conducted in accordance with the principles of the Declaration of Helsinki. All research protocols associated with the NHANES were reviewed and approved by the Ethics Review Board of the National Center for Health Statistics (NCHS). As this is a secondary analysis of publicly available data, no additional clinical trial registration was required.

## Consent

All participants provided written informed consent.

## Conflicts of Interest

The authors declare no conflicts of interest.

## Supporting information


**Figure S1:** SMOTE imputation results.

**Figure S2:** Line plot showing score changes of different features during Boruta selection.

**Figure S3:** LIME algorithm interpretation of the best‐performing machine learning model with combined demographic characteristics and dietary nutrients.

**Figure S4:** LIME algorithm interpretation of the best‐performing machine learning model using dietary nutrients alone.

**Figure S5:** Heatmaps illustrating the performance comparison of six machine learning models under different conditions in the full population.

**Figure S6:** Heatmaps comparing the performance of six machine learning models reconstructed after adjustment using the residual method, along with calibration curves and decision curve analysis (DCA) for the original models.

**Figure S7:** Heatmaps showing the performance comparison of six machine learning models under conditions combining demographic characteristics and dietary nutrients after excluding participants with known cardiovascular disease (CVD) or chronic kidney disease (CKD), and after imputing missing data.

**Figure S8:** Time‐series validation assessing model stability.

**Table S1:** Definitions and diagnostic criteria for CKM staging.

## Data Availability

The datasets generated and/or analyzed during the current study are freely available in the NHANES website (https://www.cdc.gov/nchs/nhanes/). Analysis code, preprocessing scripts, and model parameters are available upon reasonable request from the corresponding author and will be deposited in a public repository (e.g., GitHub) upon publication.
