# Supplementary figures and images for: Interpretable Prediction of Late‐Stage CKM Syndrome Association From Dietary Nutrients in Accelerated Aging Using SHAP and LIME
Source: Food Sci Nutr. 2026 Feb 17;14(2):e71547. doi: 10.1002/fsn3.71547 (PMC12913708; doi:10.1002/fsn3.71547)

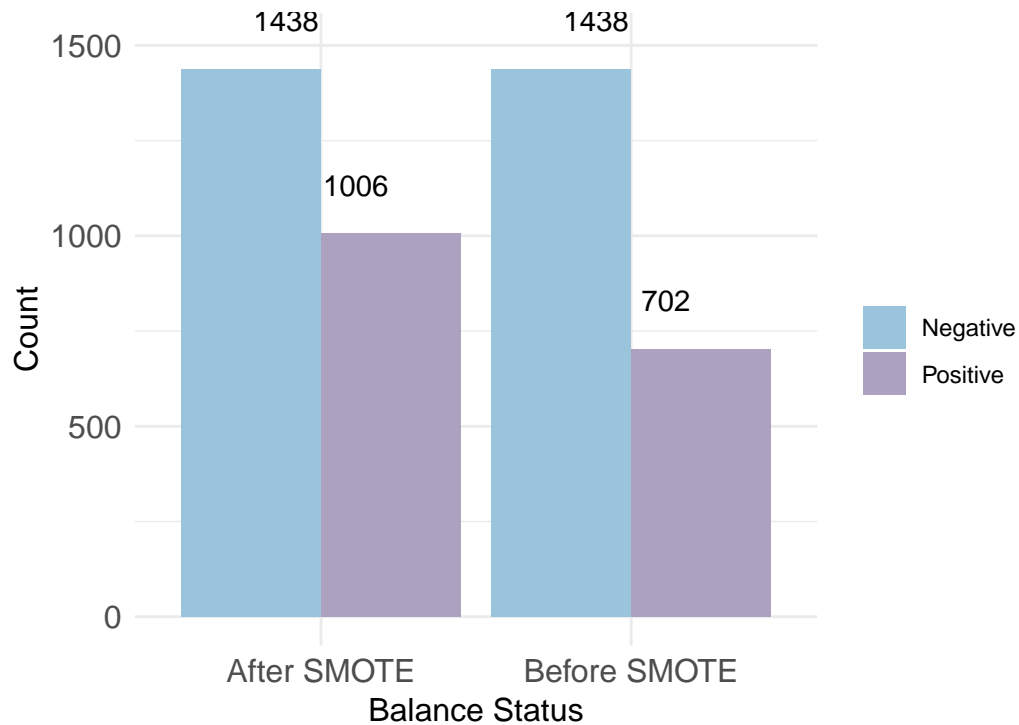

Supplement: Supplementary file 1 — Figure S1: SMOTE imputation results. [file FSN3-14-e71547-s001.pdf]

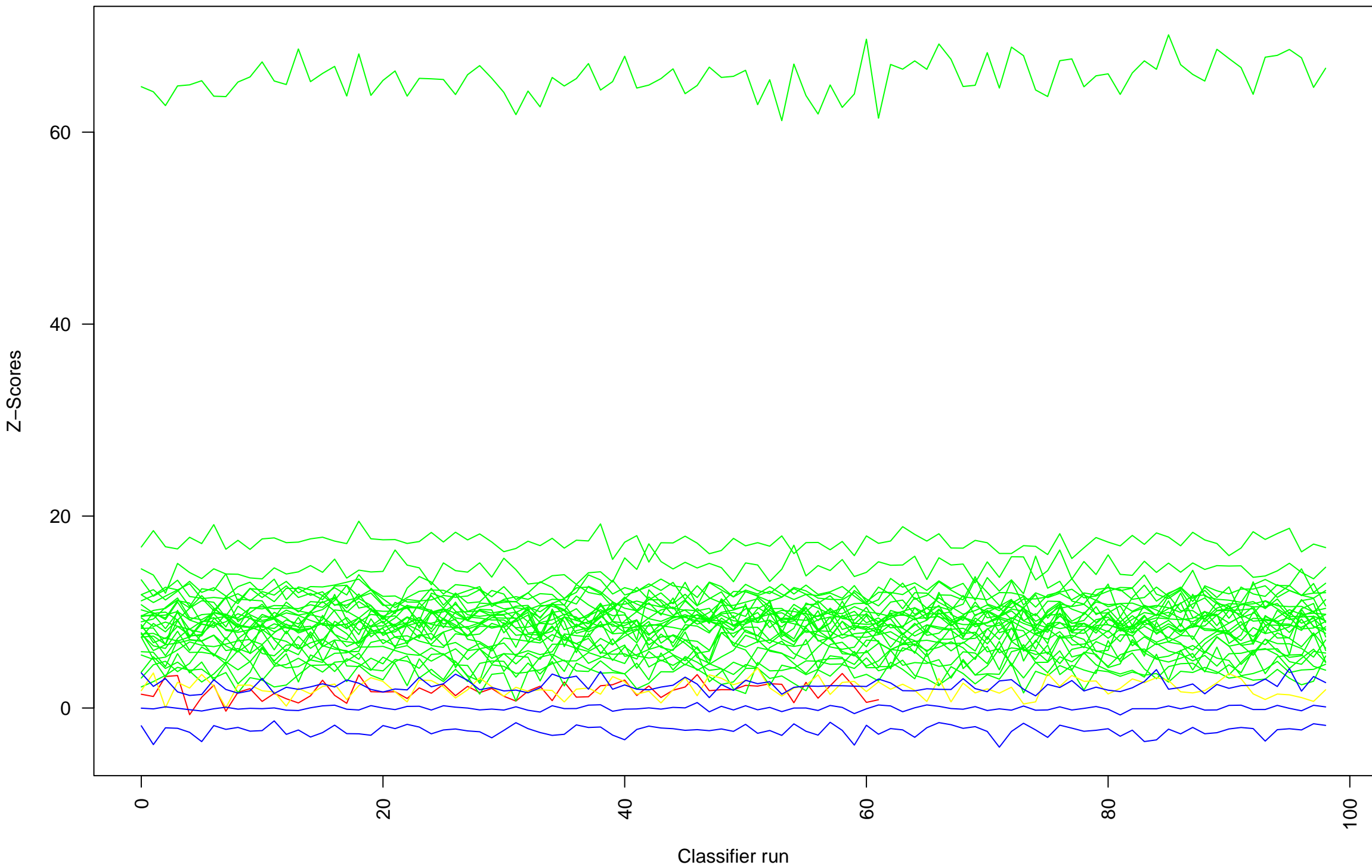

Supplement: Supplementary file 2 — Figure S2: Line plot showing score changes of different features during Boruta selection. [file FSN3-14-e71547-s008.pdf]

Machine Learning Model

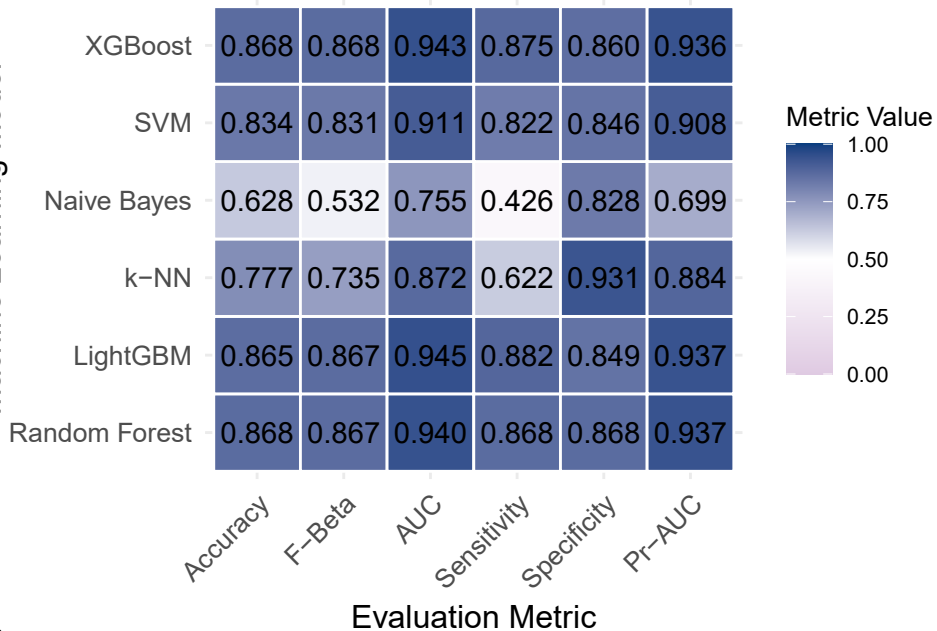

A

Machine Learning Model

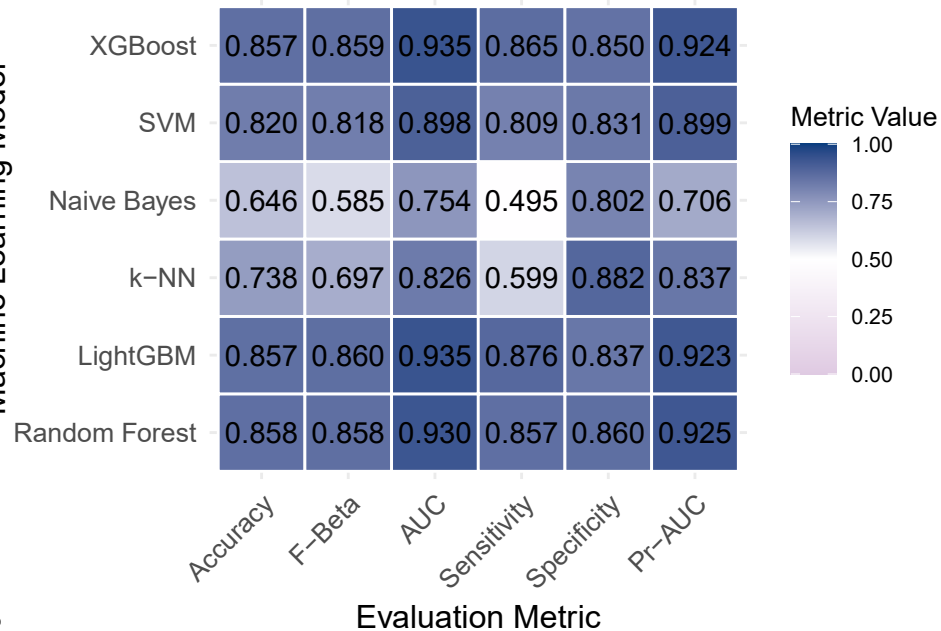

B

Brier=0.096

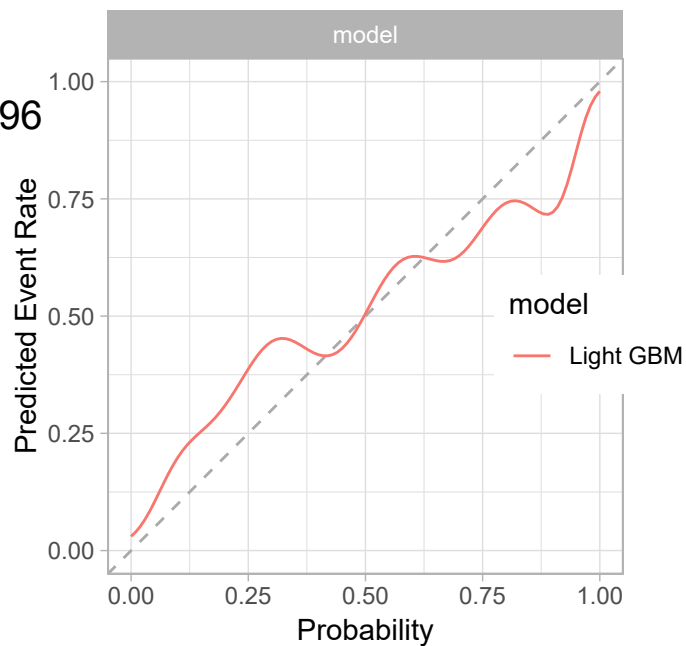

C

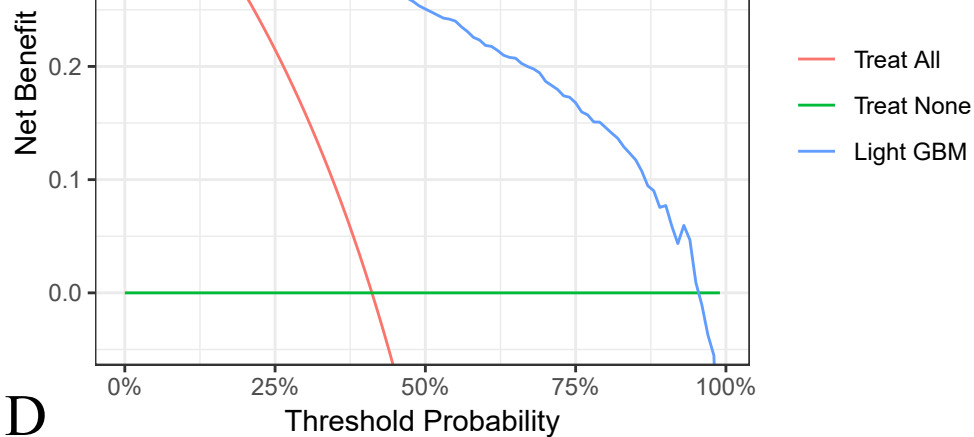

D

Supplement: Supplementary file 6 — Figure S6: Heatmaps comparing the performance of six machine learning models reconstructed after adjustment using the residual method, along with calibration curves and decision curve analysis (DCA) for the original models. [file FSN3-14-e71547-s007.pdf]

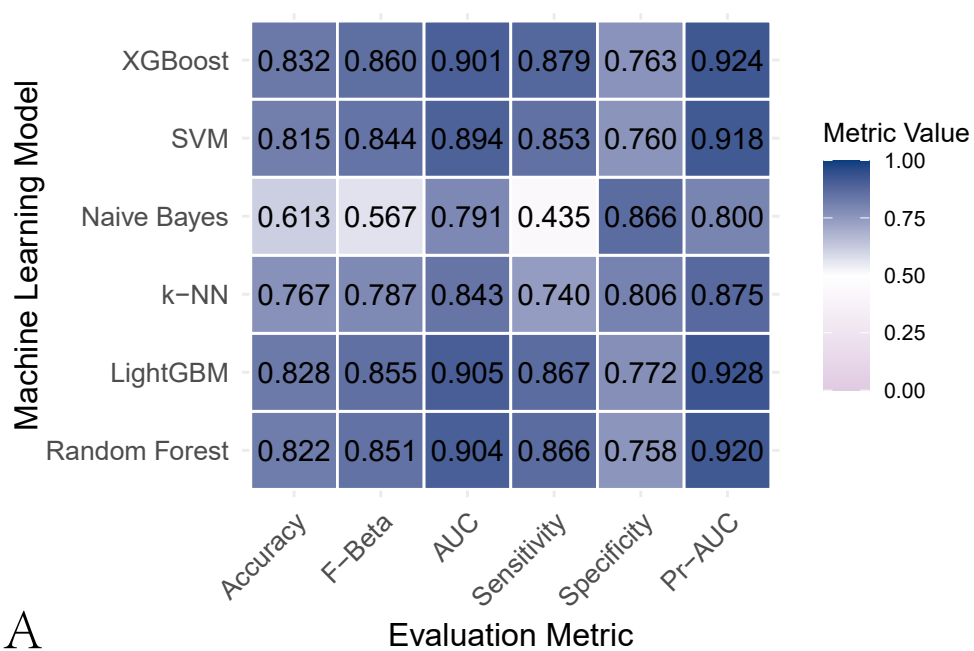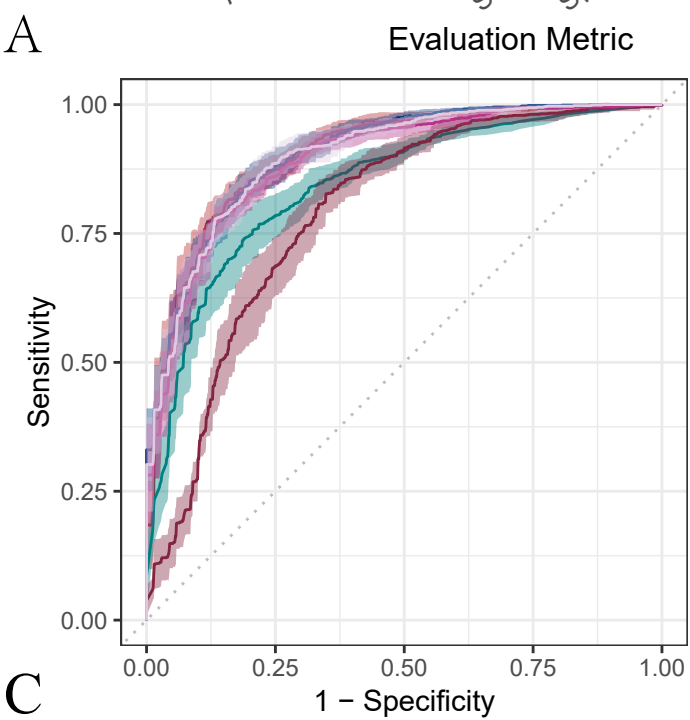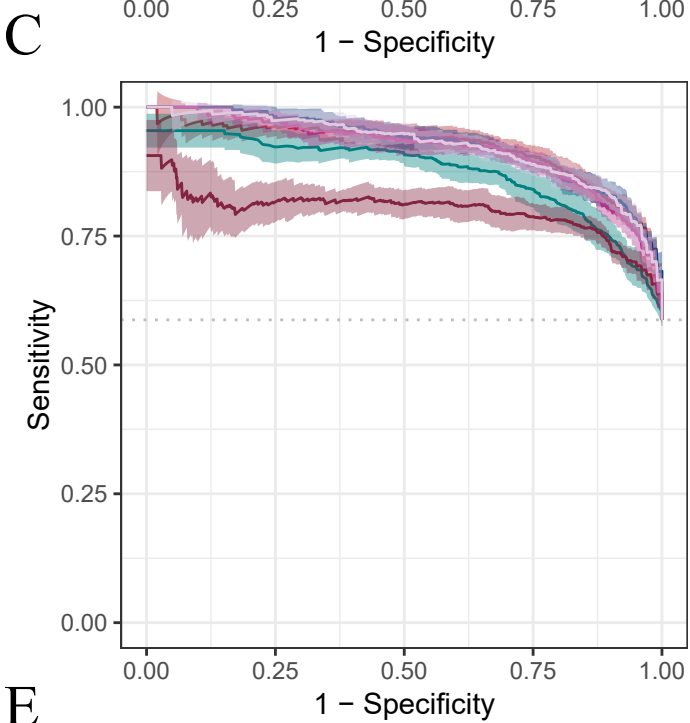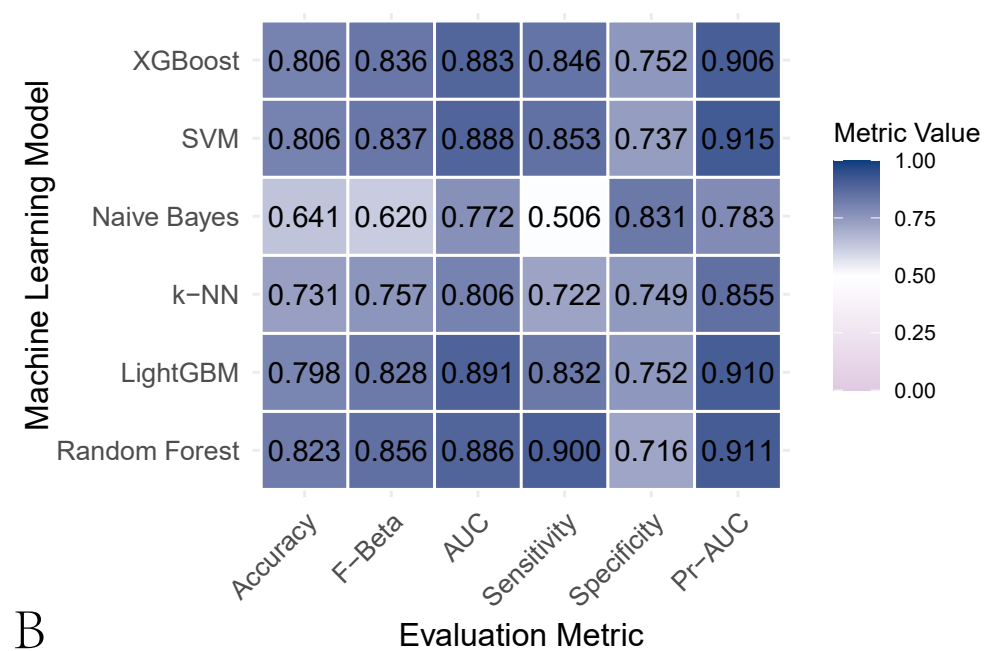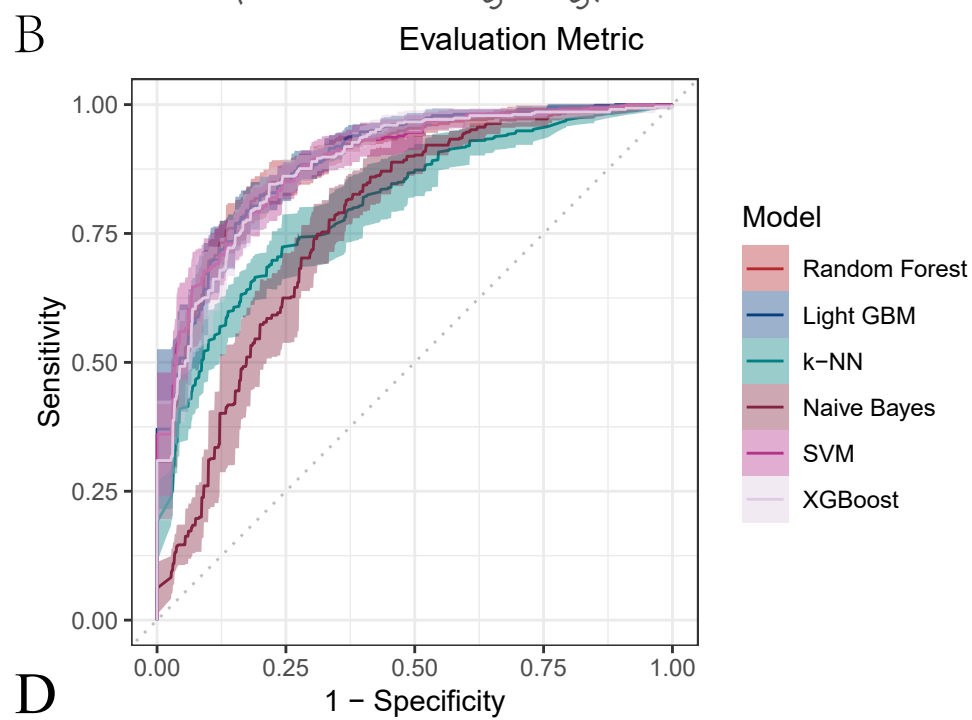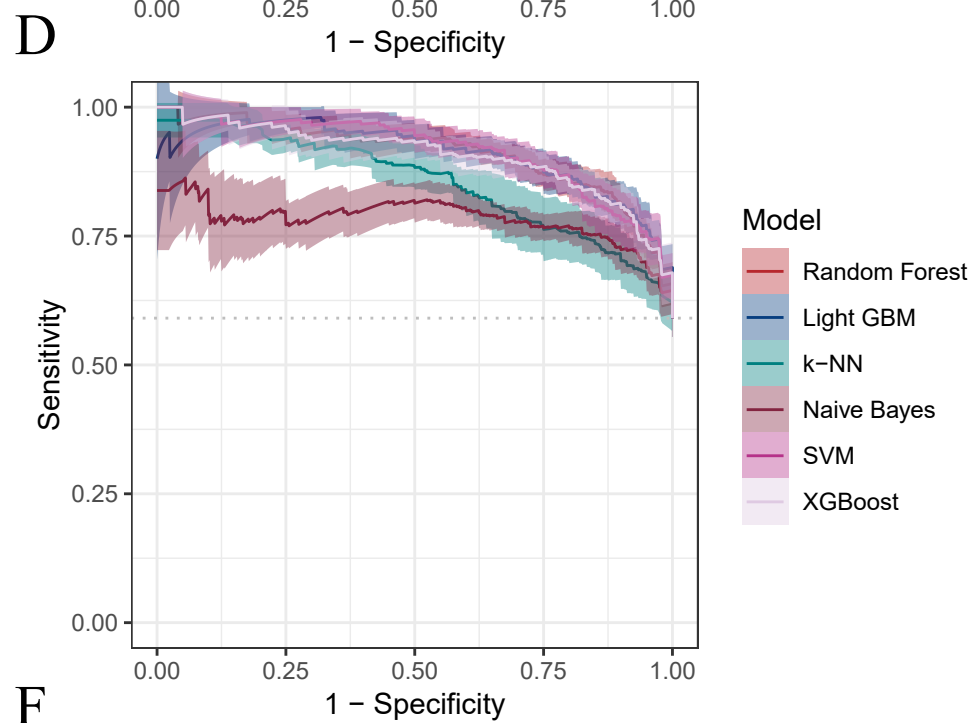

Supplement: Supplementary file 8 — Figure S8: Time‐series validation assessing model stability. [file FSN3-14-e71547-s009.pdf]
