# Supplementary material for: Interpretable Prediction of Late‐Stage CKM Syndrome Association From Dietary Nutrients in Accelerated Aging Using SHAP and LIME
Source: Food Sci Nutr. 2026 Feb 17;14(2):e71547. doi: 10.1002/fsn3.71547 (PMC12913708; doi:10.1002/fsn3.71547)
Supplement: Supplementary file 4 — Figure S4: LIME algorithm interpretation of the best‐performing machine learning model using dietary nutrients alone. [file FSN3-14-e71547-s003.pdf]

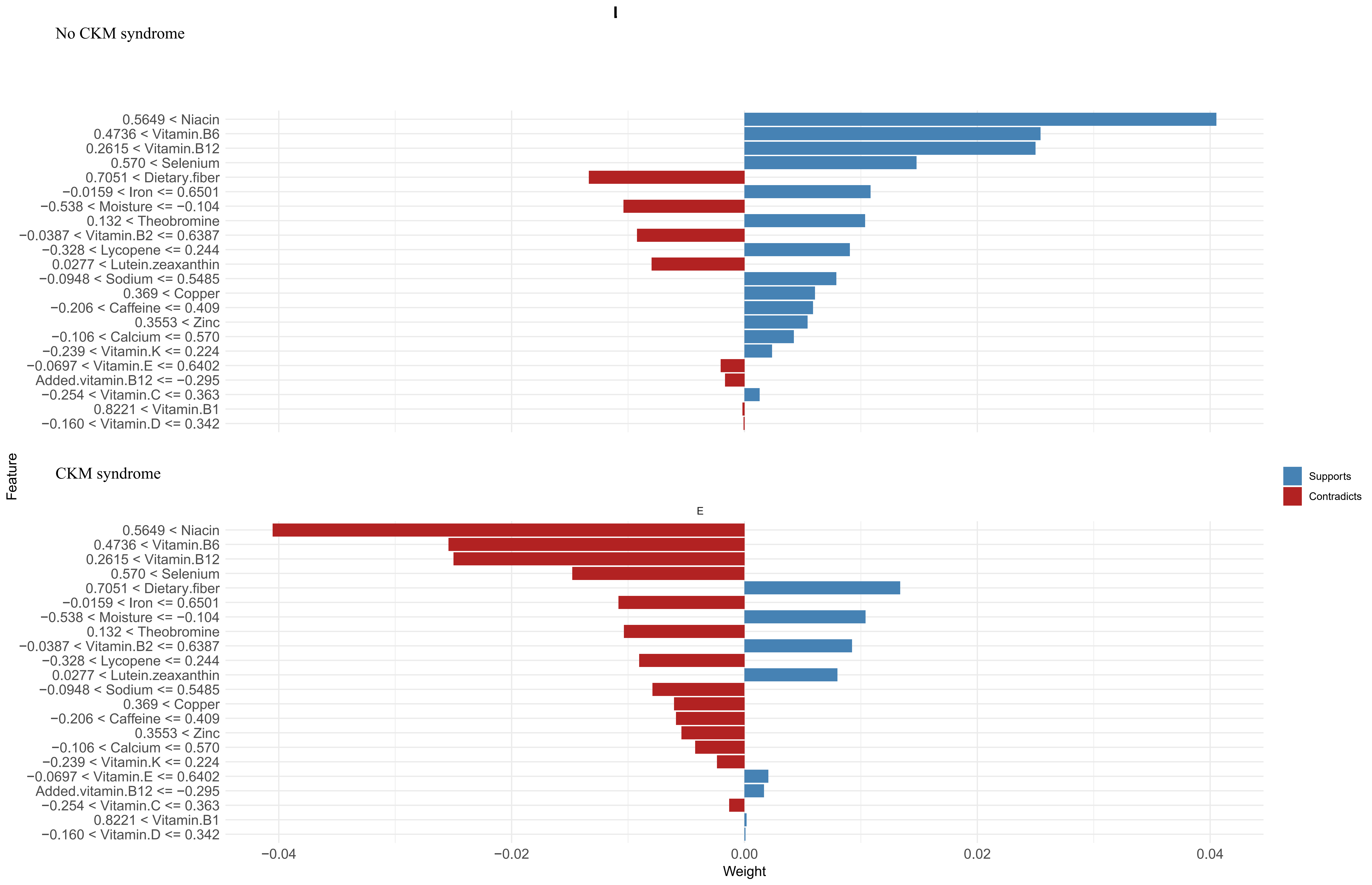

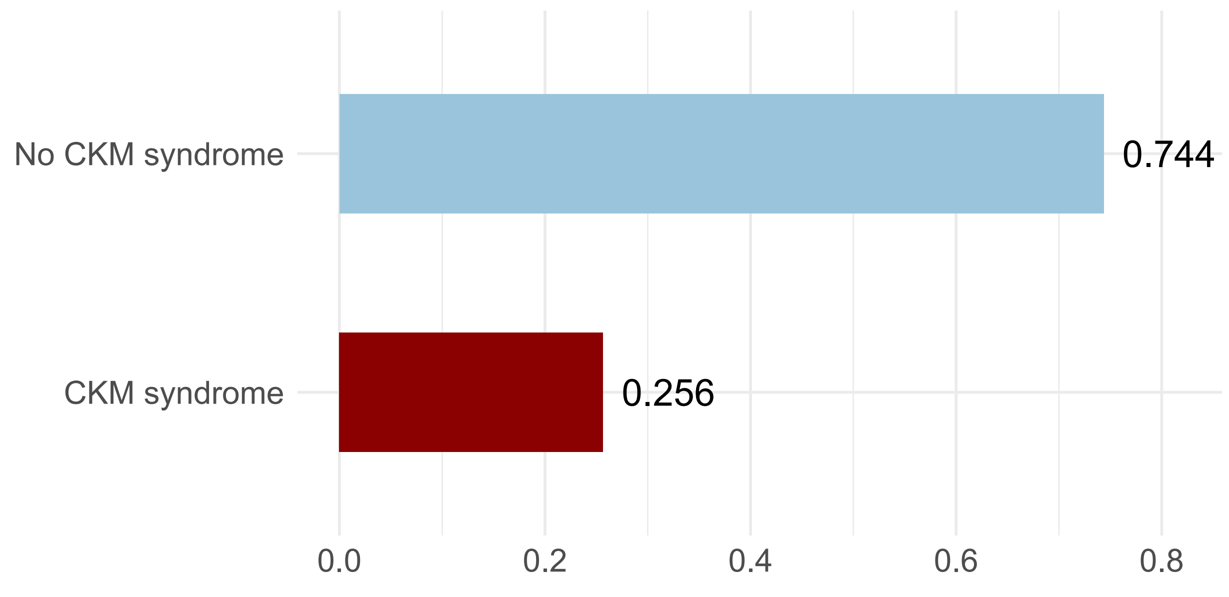

No CKM syndrome

Feature	Value
Niacin	0.81
Vitamin.B6	1.13
Vitamin.B12	0.31
Selenium	0.75
Dietary.fiber	0.76
Iron	0.46
Moisture	−0.45
Theobromine	0.28
Vitamin.B2	0.15
Lycopene	−0.31

Status: Header, Positive Contribution, Negative Contribution

CKM syndrome

Feature	Value
Niacin	0.81
Vitamin.B6	1.13
Vitamin.B12	0.31
Selenium	0.75
Dietary.fiber	0.76
Iron	0.46
Moisture	−0.45
Theobromine	0.28
Vitamin.B2	0.15
Lycopene	−0.31

Status: Header, Positive Contribution, Negative Contribution
