# Supplementary material for: Interpretable Prediction of Late‐Stage CKM Syndrome Association From Dietary Nutrients in Accelerated Aging Using SHAP and LIME
Source: Food Sci Nutr. 2026 Feb 17;14(2):e71547. doi: 10.1002/fsn3.71547 (PMC12913708; doi:10.1002/fsn3.71547)
Supplement: Supplementary file 5 — Figure S5: Heatmaps illustrating the performance comparison of six machine learning models under different conditions in the full population. [file FSN3-14-e71547-s005.pdf]

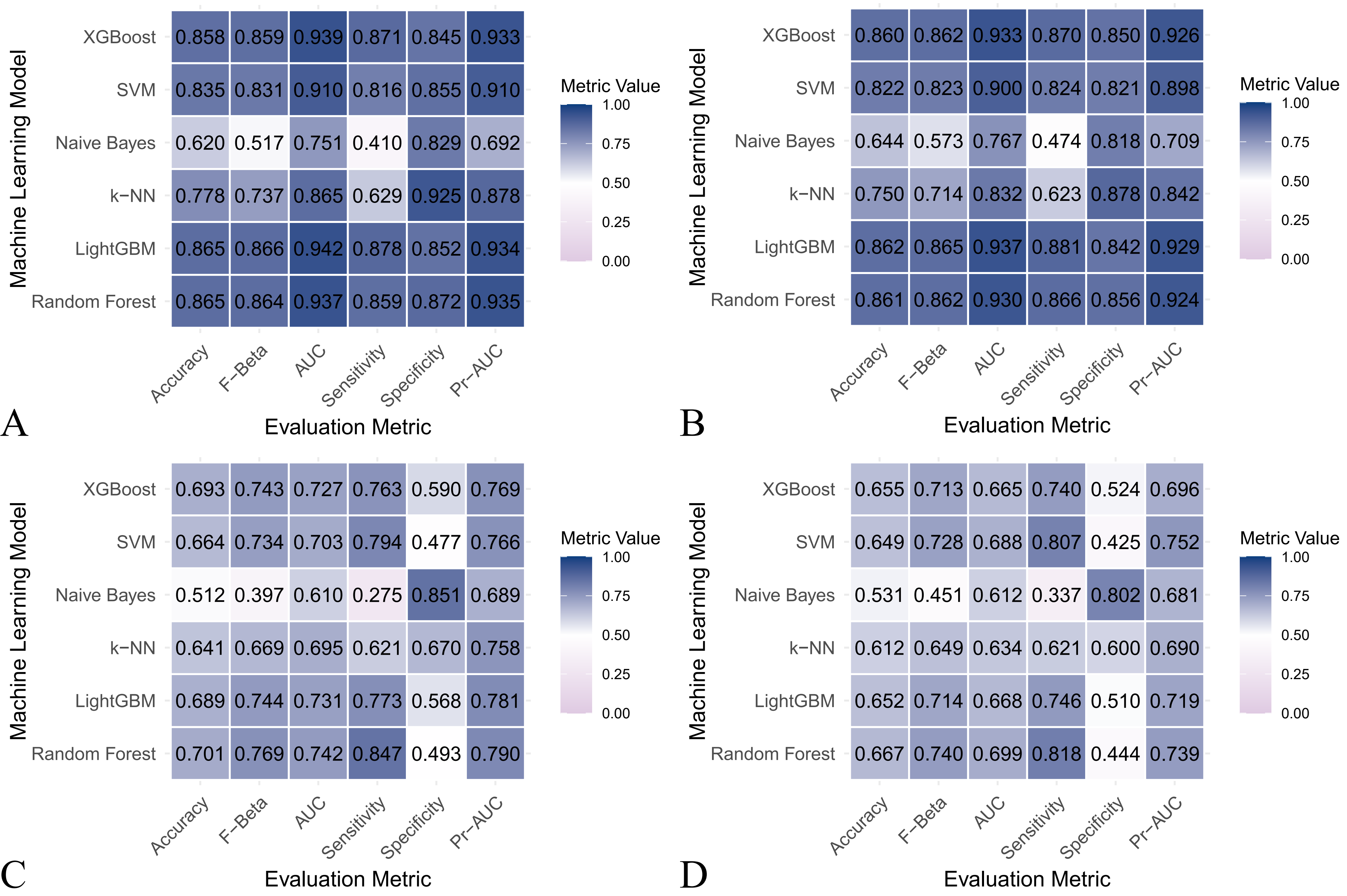
A
Machine Learning Model
XGBoost 0.858 0.859 0.939 0.871 0.845 0.933
SVM 0.835 0.831 0.910 0.816 0.855 0.910
Naive Bayes 0.620 0.517 0.751 0.410 0.829 0.692
k−NN 0.778 0.737 0.865 0.629 0.925 0.878
LightGBM 0.865 0.866 0.942 0.878 0.852 0.934
Random Forest 0.865 0.864 0.937 0.859 0.872 0.935
Accuracy F−Beta AUC Sensitivity Specificity Pr−AUC
Evaluation Metric
Metric Value
1.00
0.75
0.50
0.25
0.00
B
Machine Learning Model
XGBoost 0.860 0.862 0.933 0.870 0.850 0.926
SVM 0.822 0.823 0.900 0.824 0.821 0.898
Naive Bayes 0.644 0.573 0.767 0.474 0.818 0.709
k−NN 0.750 0.714 0.832 0.623 0.878 0.842
LightGBM 0.862 0.865 0.937 0.881 0.842 0.929
Random Forest 0.861 0.862 0.930 0.866 0.856 0.924
Accuracy F−Beta AUC Sensitivity Specificity Pr−AUC
Evaluation Metric
Metric Value
1.00
0.75
0.50
0.25
0.00
C
Machine Learning Model
XGBoost 0.693 0.743 0.727 0.763 0.590 0.769
SVM 0.664 0.734 0.703 0.794 0.477 0.766
Naive Bayes 0.512 0.397 0.610 0.275 0.851 0.689
k−NN 0.641 0.669 0.695 0.621 0.670 0.758
LightGBM 0.689 0.744 0.731 0.773 0.568 0.781
Random Forest 0.701 0.769 0.742 0.847 0.493 0.790
Accuracy F−Beta AUC Sensitivity Specificity Pr−AUC
Evaluation Metric
Metric Value
1.00
0.75
0.50
0.25
0.00
D
Machine Learning Model
XGBoost 0.655 0.713 0.665 0.740 0.524 0.696
SVM 0.649 0.728 0.688 0.807 0.425 0.752
Naive Bayes 0.531 0.451 0.612 0.337 0.802 0.681
k−NN 0.612 0.649 0.634 0.621 0.600 0.690
LightGBM 0.652 0.714 0.668 0.746 0.510 0.719
Random Forest 0.667 0.740 0.699 0.818 0.444 0.739
Accuracy F−Beta AUC Sensitivity Specificity Pr−AUC
Evaluation Metric
Metric Value
1.00
0.75
0.50
0.25
0.00
