# Supplementary material for: Interpretable Prediction of Late‐Stage CKM Syndrome Association From Dietary Nutrients in Accelerated Aging Using SHAP and LIME
Source: Food Sci Nutr. 2026 Feb 17;14(2):e71547. doi: 10.1002/fsn3.71547 (PMC12913708; doi:10.1002/fsn3.71547)
Supplement: Supplementary file 7 — Figure S7: Heatmaps showing the performance comparison of six machine learning models under conditions combining demographic characteristics and dietary nutrients after excluding participants with known cardiovascular disease (CVD) or chronic kidney disease (CKD), and after imputing missing data. [file FSN3-14-e71547-s002.pdf]

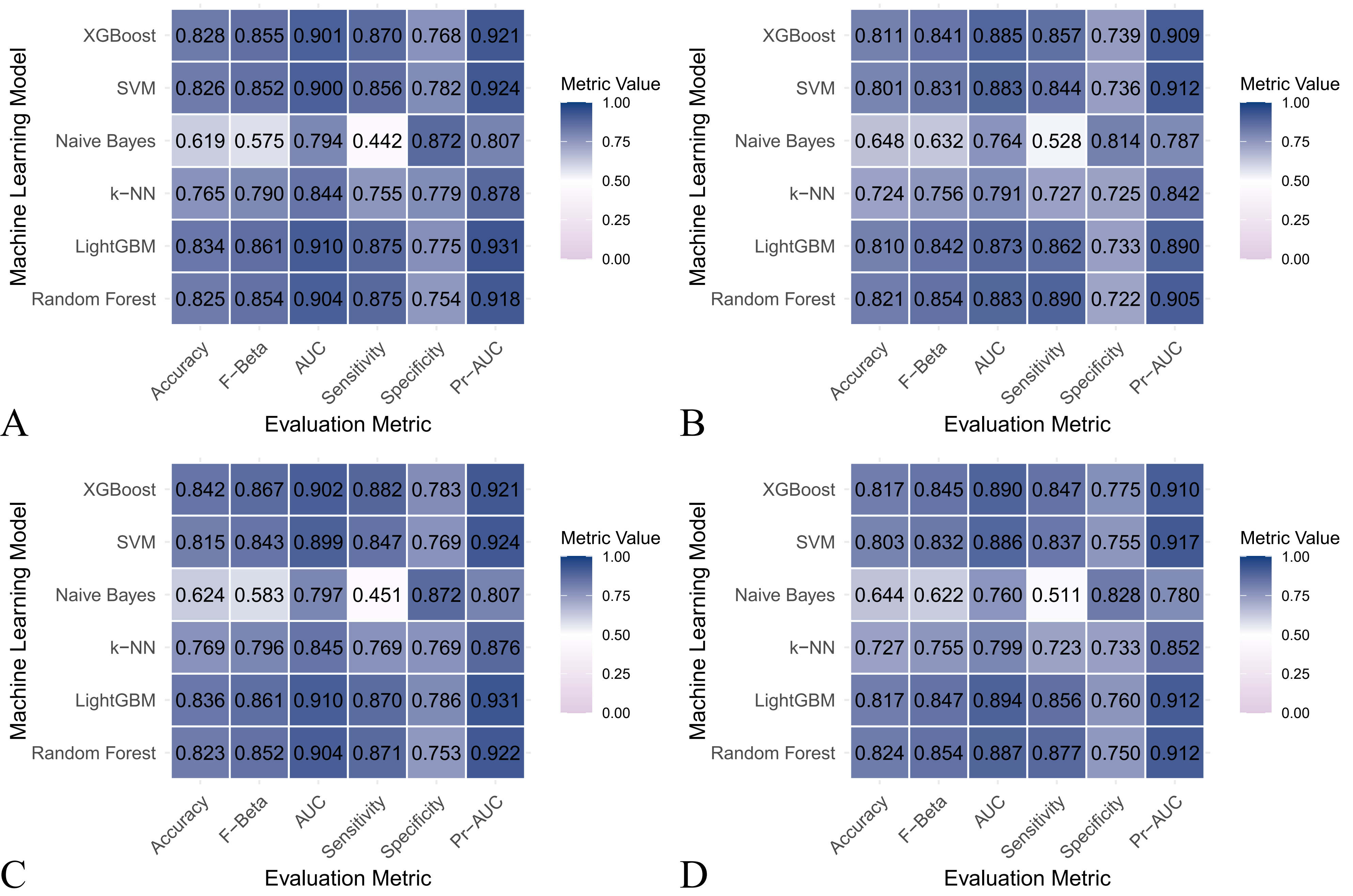
A
Machine Learning Model
XGBoost 0.828 0.855 0.901 0.870 0.768 0.921
SVM 0.826 0.852 0.900 0.856 0.782 0.924
Naive Bayes 0.619 0.575 0.794 0.442 0.872 0.807
k−NN 0.765 0.790 0.844 0.755 0.779 0.878
LightGBM 0.834 0.861 0.910 0.875 0.775 0.931
Random Forest 0.825 0.854 0.904 0.875 0.754 0.918
Accuracy F−Beta AUC Sensitivity Specificity Pr−AUC
Evaluation Metric
Metric Value
1.00
0.75
0.50
0.25
0.00
B
Machine Learning Model
XGBoost 0.811 0.841 0.885 0.857 0.739 0.909
SVM 0.801 0.831 0.883 0.844 0.736 0.912
Naive Bayes 0.648 0.632 0.764 0.528 0.814 0.787
k−NN 0.724 0.756 0.791 0.727 0.725 0.842
LightGBM 0.810 0.842 0.873 0.862 0.733 0.890
Random Forest 0.821 0.854 0.883 0.890 0.722 0.905
Accuracy F−Beta AUC Sensitivity Specificity Pr−AUC
Evaluation Metric
Metric Value
1.00
0.75
0.50
0.25
0.00
C
Machine Learning Model
XGBoost 0.842 0.867 0.902 0.882 0.783 0.921
SVM 0.815 0.843 0.899 0.847 0.769 0.924
Naive Bayes 0.624 0.583 0.797 0.451 0.872 0.807
k−NN 0.769 0.796 0.845 0.769 0.769 0.876
LightGBM 0.836 0.861 0.910 0.870 0.786 0.931
Random Forest 0.823 0.852 0.904 0.871 0.753 0.922
Accuracy F−Beta AUC Sensitivity Specificity Pr−AUC
Evaluation Metric
Metric Value
1.00
0.75
0.50
0.25
0.00
D
Machine Learning Model
XGBoost 0.817 0.845 0.890 0.847 0.775 0.910
SVM 0.803 0.832 0.886 0.837 0.755 0.917
Naive Bayes 0.644 0.622 0.760 0.511 0.828 0.780
k−NN 0.727 0.755 0.799 0.723 0.733 0.852
LightGBM 0.817 0.847 0.894 0.856 0.760 0.912
Random Forest 0.824 0.854 0.887 0.877 0.750 0.912
Accuracy F−Beta AUC Sensitivity Specificity Pr−AUC
Evaluation Metric
Metric Value
1.00
0.75
0.50
0.25
0.00
