# Supplementary material for: Interpretable Prediction of Late‐Stage CKM Syndrome Association From Dietary Nutrients in Accelerated Aging Using SHAP and LIME
Source: Food Sci Nutr. 2026 Feb 17;14(2):e71547. doi: 10.1002/fsn3.71547 (PMC12913708; doi:10.1002/fsn3.71547)
Supplement: Supplementary file 9 — Table S1: Definitions and diagnostic criteria for CKM staging. [file FSN3-14-e71547-s006.docx]

**Supplementary Table 1** Definitions and diagnostic criteria for CKM staging

| CKM stages | Definition | Criterion | Variables and diagnostic thresholds for CKM |
| --- | --- | --- | --- |
| Stage 0: No CKM risk factors | Individuals with normal BMI and waist circumference, normoglycemia, normotension, a normal lipid profile, and no evidence of CKD or subclinical or clinical CVD | All criteria are met | 1. BMI < 25 kg/m^2^ (or < 23 kg/m^2^ if Asian ancestry); 2. Waist circumference < 88/102 cm in female/male (or if Asian ancestry < 80/90 cm in female/male); 3. Fasting blood glucose < 100 mg/dL and HbA1c < 5.7% and without self-reported diagnosis of diabetes, use of insulin, or oral hypoglycemic agents; 4. SBP < 130 mmHg and DBP < 80 mmHg, without self-reported diagnosis of hypertension or use of antihypertensive medications; 5. HDL cholesterol < 50/40 mg/dL in female/male, and triglycerides < 150 mg/dL 6. Low-risk CKD in KDIGO classification according to eGFR and UACR: UACR < 30 mg/g and eGFR ≥ 60 mL/min/1.73 m^2^; 7. Predicted 10-year CVD risk < 20%; 8. No clinical CVD; |
| Stage 1: Excess or dysfunctional adiposity | Individuals with overweight/obesity, abdominal obesity, or dysfunctional adipose tissue, without the presence of other metabolic risk factors or CKD | Any of the three criteria is met | 1. Overweight/obesity; 2. Abdominal obesity; 3. Prediabetes |
|  |  | All criteria are met | 1. SBP < 130 mmHg and DBP < 80 mmHg without self-reported diagnosis of hypertension or use of antihypertensive medications; 2. HDL cholesterol < 50/40 mg/dL in female/male and triglycerides < 150 mg/dL; 3. Low-risk CKD in KDIGO classification according to eGFR and UACR: UACR < 30 mg/g and eGFR ≥ 60 mL/min/1.73 m^2^; 4. Predicted 10-year CVD risk < 20%; 5. No clinical CVD; |
| Stage 2: Metabolic risk factors and CKD | Individuals with metabolic risk factors (hypertriglyceridemia, hypertension, MetS, or diabetes) or CKD | Any of the five criteria is met | 1. Hypertriglyceridemia; 2. Hypertension; 3. Diabetes; 4. MetS; 5. Moderate-to-high-risk CKD in KDIGO classification |
|  |  | All criteria are met | 1. No very high-risk CKD in KDIGO classification; 2. Predicted 10-year CVD risk < 20%; 3. No clinical CVD; |
| Stage 3: Subclinical CVD in CKM | Subclinical CVD among individuals with excess/dysfunctional adiposity, other metabolic risk factors, or CKD | Any of the two criteria is met | 1. Very high-risk CKD in KDIGO classification; 2. Predicted 10-year CVD risk ≥ 20% |
|  |  | Any of the eight criteria is met | 1. Overweight/obesity; 2. Abdominal obesity; 3. Prediabetes; 4. Hypertriglyceridemia; 5. Hypertension; 6. Diabetes; 7. MetS; 8. Moderate-to-high-risk CKD in KDIGO classification |
|  |  | The criterion is met | No clinical CVD |
| Stage 4: Clinical CVD in CKM | Clinical CVD among individuals with excess/dysfunctional adiposity, other metabolic risk factors, or CKD | The criterion is met | Clinical CVD |
|  |  | Any of the nine criteria is met | 1. Overweight/obesity; 2. Abdominal obesity; 3. Prediabetes; 4. Hypertriglyceridemia; 5. Hypertension; 6. Diabetes; 7. MetS; 8. Moderate-to-high-risk CKD in KDIGO classification; 9. Very high-risk CKD in KDIGO classification |

**Abbreviations**: BMI, body mass index; CKD, chronic kidney disease; CKM, cardiometabolic–kidney; CVD, cardiovascular disease; DBP, diastolic blood pressure; eGFR, estimated glomerular filtration rate; HbA1c, glycated hemoglobin; HDL, high-density lipoprotein; KDIGO, Kidney Disease: Improving Global Outcomes; MetS, metabolic syndrome; SBP, systolic blood pressure; UACR, urine albumin-to-creatinine ratio.
