# Supplementary material for: Interpretable Prediction of Late‐Stage CKM Syndrome Association From Dietary Nutrients in Accelerated Aging Using SHAP and LIME
Source: Food Sci Nutr. 2026 Feb 17;14(2):e71547. doi: 10.1002/fsn3.71547 (PMC12913708; doi:10.1002/fsn3.71547)
Supplement: Supplementary file 3 — Figure S3: LIME algorithm interpretation of the best‐performing machine learning model with combined demographic characteristics and dietary nutrients. [file FSN3-14-e71547-s004.pdf]

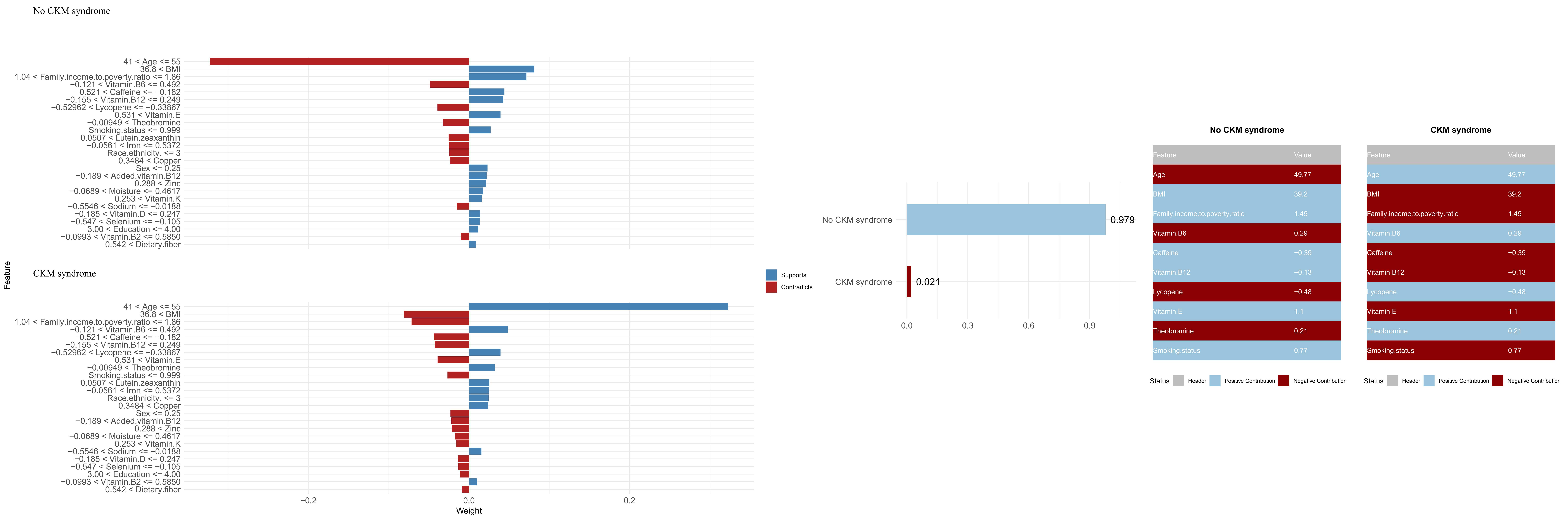
No CKM syndrome
41 < Age <= 55
36.8 < BMI
1.04 < Family.income.to.poverty.ratio <= 1.86
−0.121 < Vitamin.B6 <= 0.492
−0.521 < Caffeine <= −0.182
−0.155 < Vitamin.B12 <= 0.249
−0.52962 < Lycopene <= −0.33867
0.531 < Vitamin.E
−0.00949 < Theobromine
Smoking.status <= 0.999
0.0507 < Lutein.zeaxanthin
−0.0561 < Iron <= 0.5372
Race.ethnicity. <= 3
0.3484 < Copper
Sex <= 0.25
−0.189 < Added.vitamin.B12
0.288 < Zinc
−0.0689 < Moisture <= 0.4617
0.253 < Vitamin.K
−0.5546 < Sodium <= −0.0188
−0.185 < Vitamin.D <= 0.247
−0.547 < Selenium <= −0.105
3.00 < Education <= 4.00
−0.0993 < Vitamin.B2 <= 0.5850
0.542 < Dietary.fiber
Feature
CKM syndrome
−0.2
0.0
0.2
Weight
Supports
Contradicts
No CKM syndrome
CKM syndrome
0.979
0.021
0.0
0.3
0.6
0.9
No CKM syndrome
Feature
Value
Age
49.77
BMI
39.2
Family.income.to.poverty.ratio
1.45
Vitamin.B6
0.29
Caffeine
−0.39
Vitamin.B12
−0.13
Lycopene
−0.48
Vitamin.E
1.1
Theobromine
0.21
Smoking.status
0.77
Status
Header
Positive Contribution
Negative Contribution
CKM syndrome
Feature
Value
Age
49.77
BMI
39.2
Family.income.to.poverty.ratio
1.45
Vitamin.B6
0.29
Caffeine
−0.39
Vitamin.B12
−0.13
Lycopene
−0.48
Vitamin.E
1.1
Theobromine
0.21
Smoking.status
0.77
Status
Header
Positive Contribution
Negative Contribution
